# Trivalent multi-epitope mRNA vaccine against norovirus, rotavirus, and adenovirus 40/41: epitope screening, molecular docking, and molecular dynamics simulation with in silico validation guided by immunoinformatics

**DOI:** 10.1186/s40249-026-01431-y

**Published:** 2026-03-27

**Authors:** Xu Wu, Yizhong Xu, Rongliu Qin, Yuying Luo, Yanqun Hou, Ziyou Zhou, Ruping Qu, Shiyang Ma, Jie Chen, Fei Zhu, Pinhua Pan

**Affiliations:** 1 Department of Respiratory Medicine, National Key Clinical Specialty, Branch of National Clinical Research Center for Respiratory Disease, Xiangya Hospital, Central South University, Changsha, Hunan China; 2 Center of Respiratory Medicine, Xiangya Hospital, Central South University, Changsha, 410008 Hunan China; 3 Clinical Research Center for Respiratory Diseases in Hunan Province, Changsha, Hunan China; 4 Hunan Engineering Research Center for Intelligent Diagnosis and Treatment of Respiratory Disease, Changsha, Hunan China; 5 National Clinical Research Center for Geriatric Disease (Xiangya Hospital), Changsha, Hunan China; 6 FuRong Laboratory, Changsha, 410078 Hunan China

**Keywords:** Norovirus, Rotavirus, Adenovirus 40/41, Multi-epitope mRNA vaccine, Immunoinformatics, Molecular dynamics simulation, Molecular docking

## Abstract

**Background:**

Diarrheal diseases constitute a major global public health threat, particularly endangering young children, the elderly, and immunocompromised individuals. Three key pathogens—norovirus, rotavirus, and adenovirus 40/41—can induce dehydration, electrolyte imbalances, and severe complications, resulting in tens of thousands of deaths annually. Conventional vaccines have inherent limitations, including relatively long development cycles and high production costs. With the deep integration of bioinformatics and immunology, immunoinformatic techniques driven by high-throughput analysis enable reliable prediction of key epitope properties such as immunogenicity and antigenicity, offering an efficient approach for multivalent vaccine development. This study aims to develop a trivalent multi-epitope mRNA vaccine targeting these three pathogens using immunoinformatic methods, providing a potential innovative strategy for the prevention and control of diarrheal diseases.

**Methods:**

The amino acid sequences corresponding to the target viral proteins were obtained from the NCBI Virus Database. Epitopes were screened and selected based on key properties including high antigenicity, non-allergenicity, and non-toxicity. Appropriate adjuvant components, along with the chosen T-lymphocyte and B-lymphocyte epitopes, were assembled using linker molecules to computationally construct the vaccine. Structural and related features of the computationally designed vaccine were analyzed using online tools. Molecular docking assays, in conjunction with molecular dynamics simulations, were performed to clarify the interaction modes and structural stability characteristics of ligand-receptor binding. mRNA sequences of the vaccine were designed through codon optimization, and their immunogenicity was ultimately assessed using immune simulations.

**Results:**

A total of 16 cytotoxic T-cell epitopes, 5 helper T-cell epitopes, and 17 linear B-cell epitopes were selected to construct the vaccine. After evaluating immunological and physicochemical properties, molecular docking and molecular dynamics simulations were performed, suggesting favorable structural stability and plausible interactions with immune receptors.

**Conclusions:**

The computationally designed vaccine in this study was predicted to exhibit favorable structural stability, potential immune activation capability, and promising broad population coverage, providing preliminary insights for the development of vaccines against multiple viral co-infections; however, its immunogenicity and safety remain to be further validated through animal model experiments.

**Supplementary Information:**

The online version contains supplementary material available at 10.1186/s40249-026-01431-y.

## Background

Diarrheal diseases remain a major global public health burden, with high occurrence rates and disease burdens persisting as threats to population health. Drawing on data provided by the World Health Organization alongside the Global Burden of Disease Study, the global annual incidence count of diarrheal disease has long stayed at 1.7–1.8 billion cases, and diarrheal conditions stand as the eighth top cause of death globally [1]. Worldwide, around 1.17 million deaths were linked to diarrheal conditions in 2021, with children under 5 years old resulting in a disproportionately heavy disease burden. They account for 52.4% of the global diarrhea-related disability-adjusted life years, equivalent to 30.9 million out of 59.0 million [2]. While this figure represents a 60.3% decrease compared with 1990, there is a notable imbalance in the geographical distribution of disease burden: low- and middle-income countries (LMICs) account for the vast majority of cases and deaths, most notably in South Asia and sub-Saharan Africa. In these regions, the diarrhea mortality rate among children under 5 years old is as high as 10–20 times greater than that in high-income countries [2, 3].

Viral diarrhea is more common than bacterial and parasitic diarrhea, accounting for 36.4% of the total incidence. Norovirus, rotavirus, and adenovirus 40/41, three core viral pathogens underlying diarrhea, pose substantial challenges in terms of their epidemiological characteristics and pathogenic mechanisms [4]. Norovirus is characterized by high infectivity and strong environmental resistance, often triggering outbreaks in collective settings such as childcare facilities and schools. Globally, norovirus is linked to approximately one-fifth of all diarrhea cases, leading to more than 200,000 annual deaths in developing nations. Additionally, it ranks as the primary driver of foodborne outbreak events across the United States [5, 6]. Although the implementation of rotavirus vaccines has reduced rotavirus incidence and mortality, rotavirus remains the primary pathogen responsible for diarrhea-related deaths among children under 5 years of age, with a notably severe impact within developing nations [7]. Adenovirus 40/41, on the other hand, can cause both diarrhea and respiratory symptoms, and it is prone to progressing to severe disease among children under 5 years of age [4]. Collectively, these three pathogens constitute the primary challenges in managing and preventing diarrheal diseases.

Vaccines are effective tools for preventing viral infections; however, vaccine development targeting these three pathogens faces significant limitations. Licensed rotavirus vaccines, while capable of reducing the rate of severe disease, exhibit insufficient cross-protection against different genotypes [8]. For norovirus, no licensed vaccine is available, and research remains in the clinical trial phase [9]. The development of adenovirus diarrhea vaccines is still in the preclinical stage [10]. Traditional vaccine development, which largely relies on inactivated/attenuated whole viruses or recombinant proteins, has inherent drawbacks: whole-virus vaccines may induce nonspecific immune responses or retain residual potential virulence; single-protein vaccines struggle to cover viral variants, resulting in limited cross-protection; and the lengthy development cycle fails to rapidly respond to viral evolution [11]. Additionally, coinfections with these three viruses are common, limiting the preventive efficacy of single-pathogen vaccines. In contrast, mRNA vaccines can circumvent these limitations and enable cost-effective, rapid, and large-scale production [12]. Therefore, the present study aimed to screen for cytotoxic T lymphocyte (CTL), helper T lymphocyte (HTL), and linear B lymphocyte (LBL) epitopes with high antigenicity, non-toxicity, and non-allergenicity. By integrating appropriate adjuvants, we aim to develop a trivalent multi-epitope mRNA vaccine targeting norovirus, rotavirus, and adenovirus 40/41.

## Methods

### Screening and identification of targeted protein sequences

#### Initial sequence retrieval and preliminary protein screening

Reference protein sequences of norovirus, rotavirus, and adenovirus 40/41 were retrieved from the NCBI Virus Database to construct an initial screening library [13, 14]. This library contained 3 norovirus-encoded proteins, 11 rotavirus-encoded proteins, and 25 major adenovirus 40/41-encoded proteins, laying a foundation for subsequent precise screening.

A four-step progressive screening strategy was adopted to systematically screen the initial protein library, with the tools, parameters, and purposes of each step as follows [15]: (1) Antigenicity screening: The VaxiJen v2.0 server was used to predict protein antigenicity [16, 17], with a screening threshold of antigenicity score > 0.4 to ensure target proteins possess the core potential to induce host-specific immune responses; (2) Toxicity and allergenicity screening: the ToxinPred v2.0 server was employed to predict and exclude proteins containing toxic domains (thresholds: 0.6) [18, 19], while the AllerTOP v2.0 server was used to identify allergenic proteins (thresholds: 0.5) [20, 21], eliminating potential safety risks in vaccine application at the source; (3) Transmembrane helix screening: the DeepTMHMM server was utilized to predict protein transmembrane structures [22, 23], and proteins with one or more transmembrane helices were excluded—such membrane-embedded structures are prone to causing issues like abnormal folding or low expression levels during subsequent recombinant expression; (4) Subcellular localization screening: The Virus-mPLoc server was used to predict protein subcellular localization [24, 25], and only proteins localized to the viral capsid, extracellular space, or cell membrane surface were retained to ensure direct recognition by the host immune system for initiating immune responses.

#### Phylogenetic analysis and core target sequence optimization

Based on the above preliminary screening results, norovirus VP1 protein, rotavirus VP4/VP6/VP7 proteins, adenovirus 40/41 hexon protein, and adenovirus 40/41 short fiber protein were ultimately identified as core target proteins. To ensure that the sequences used for epitope screening cover viral evolutionary variations and possess broad representativeness, this study further conducted phylogenetic analysis and representative sequence screening, following the specific workflow: First, sequences of each target protein across different subtypes/strains were retrieved from the NCBI Virus Database. After a series of quality control processes (including sequence deduplication, removal of fragments shorter than 95% of the reference sequence length, exclusion of non-human-derived sequences, and deletion of sequences with the unknown amino acid "X"), phylogenetic trees were constructed for the qualified sequences of each subtype. The specific method was as follows: Protein sequences were clustered using CD-HIT at 100% sequence identity to remove redundant sequences. Sequences were aligned using MAFFT v7.407 with the L-INS-i strategy under the default maximum iteration setting of 1000 for accurate alignment [26]. A maximum-likelihood phylogenetic tree was reconstructed using IQ-TREE v2 with automated model selection and 1000 ultrafast bootstrap replicates to assess branch support [27]. Finally, the phylogenetic tree was visualized using the iTOL server [28].

The screening of representative strains was based on the core principle of "high coverage". The specific method involved: For each viral subtype, a single representative (medoid) sequence was then selected for each subtype as the tip in the tree that minimizes the sum of patristic distances to all other tips, thereby providing a robust reference that minimizes the total evolutionary distance to the entire set of sequences for downstream analyses. This medoid selection was implemented in Python utilizing the ETE toolkit for tree parsing and patristic distance calculations [29].

### Epitope prediction and filtering

#### T-cell epitope prediction and filtering

The Immune Epitope Database (IEDB) is an open-access resource that compiles empirical data on antibody epitopes and T-cell epitopes, with characterization spanning humans and various other animal species, covering infectious diseases, allergies, and autoimmune diseases. In this study, when predicting T lymphocyte epitopes, two tools available via the Next-generation IEDB tools were employed: the MHC Class I Tools Suite for predicting CTL epitopes, and the MHC Class II Tools Suite for predicting HTL epitopes [30, 31].

To screen and validate CTL epitopes, two algorithms in the IEDB MHC Class I Tools Suite were used to analyze MHC-I-peptide binding: NetMHCpan 4.1 EL (Eluted Ligand assay), where epitopes with a rank percentile < 0.5% were defined as strong binders, and NetMHCpan 4.1 BA (Binding Affinity assay), where epitopes with affinity < 50 nM were classified as high-affinity binders. A panel of 27 human leukocyte antigen (HLA)-I alleles was selected, with peptide length set to 9 amino acids; only epitopes meeting both EL and BA criteria were retained. Epitopes were further filtered by the MHC-I Processing Tool (comprehensive processing score > 0) and the Class I pMHC Immunogenicity tool (immunogenicity score > 0). For safety assessment, antigenicity was analyzed via the VaxiJen v2.0 server (score > 0.4), allergenicity via the Algpred v2.0 server (threshold < 0.3) [32, 33], and toxicity via the ToxinPred v3.0 server (threshold < 0.38) [34, 35]. Only non-allergenic, non-toxic, and antigenic CTL epitopes were selected for further analyses.

For HTL epitope prediction, the MHC Class II Tools Suite was employed, with two integrated algorithms for binding screening: NetMHCIIpan 4.1 EL, where epitopes with a rank percentile < 2% were defined as strong binders, and NetMHCIIpan 4.1 BA, where epitopes with affinity < 50 nM were classified as high-affinity binders. To ensure result generalizability, 27 population-representative HLA-II alleles were included. HTL epitopes were filtered using the same safety criteria as CTL epitopes (VaxiJen v2.0, Algpred v2.0, ToxinPred v3.0). Additionally, the IFNepitope server [36, 37] was used to predict epitope-induced IFN-γ production, and the IL4pred server [38, 39] to assess IL-4 induction ability.

#### Linear B-cell epitope prediction and filtering

LBL epitopes, as key immunologically active regions on antigenic proteins, specifically bind B-cell receptors and antibodies to initiate the host’s humoral immune reaction, serving as an initiating step for antibody-mediated protection. Unlike conformation-dependent conformational epitopes, LBL epitopes are easier to predict and screen via sequence analysis tools.

To screen for potential LBL epitopes in the target protein accurately, this study utilized the ABCpred server [40, 41] for prediction and screening. The server’s model is built on a recurrent neural network trained on extensive known LBL epitope datasets, which has a robust ability to recognize sequence features. The predictive parameters included: a 16-amino-acid window and a 0.51 threshold—these parameters have been validated to achieve 65.93% accuracy, balancing detection specificity and potential epitope identification. After the preliminary prediction of linear B-cell epitopes, candidate peptides underwent multidimensional quality assessment via field-recognized tools, specifically by assessing antigenicity via the VaxiJen v2.0 server, allergenicity via the Algpred v2.0 server, and toxicity via the ToxinPred v3.0 server.

#### Secondary validation of epitope-MHC molecule binding

To further ensure that the multi-epitope mRNA vaccine can simultaneously elicit immune responses from CD8⁺ cytotoxic T cells and CD4⁺ helper T cells, MixMHCpred v3.0 and MixMHC2pred v2.0.2 servers were employed for secondary validation of MHC class I and class II molecule-binding epitope predictions, respectively [42–44]. Specifically, in MHC class I epitope prediction, 27 high-frequency HLA-I alleles prevalent in global populations were selected, with the core screening criteria defined as a best-allele percentile rank (%Rank_bestAllele) < 0.5% and a positive binding score (Score_bestAllele), whereas %Rank_bestAllele < 1% served as an auxiliary criterion, and broad-spectrum epitopes able to bind multiple alleles were prioritized for retention. For the prediction of MHC-II epitopes, this study selected 27 full-spectrum HLA class II alleles. The core screening criteria are defined as follows: epitopes with an optimal percentile rank (% Rank_best) < 2% and subtype specificity (SubSpec_best) = 1 are designated as specific high-quality epitopes; meanwhile, epitopes with % Rank_best < 20% are defined as broad-spectrum screening epitopes.

### Analysis of epitope conservancy

To assess the conservancy of screened epitopes within their respective subtypes, this study used the IEDB Epitope Conservancy Analysis Tool for systematic evaluation [45, 46]. This tool quantifies epitope conservancy via core metrics (amino acid identity, entropy) after multiple sequence alignment of target antigen homologs across strains/subtypes. For example, conservancy of epitopes from the GI.6 subtype VP1 protein was calculated in the GI.6 subtype sequence library built in Sect. "Phylogenetic analysis and core target sequence optimization". Circular heatmaps were employed to visualize the conservancy distribution of each epitope across its corresponding subtype for clear result presentation.

For secondary validation of epitope conservancy and homology, all candidate epitopes were verified using the NCBI BLASTp server. For conservancy checks, epitopes were aligned against the NCBI non-redundant (nr) database with the target pathogen set as the species filter, using parameters: E-value ≤ 1.00E-3, Query Coverage ≥ 95%, sequence identity ≥ 95% to evaluate conservancy in circulating strains. For homology validation, epitopes were aligned against the Human RefSeq database (Homo sapiens, taxid: 9606); a human-derived E-value > 0.05 was set as the safety threshold to eliminate autoimmune reaction risk. Only epitopes passing all verifications were included in the core candidate set.

### Evaluating peptide-HLA allele binding via molecular docking

We retrieved MHC molecule crystallographic structures from the RCSB Protein Data Bank [47, 48]; in parallel, we constructed homology models for these molecules via the SWISS-MODEL server [49, 50]. Molecular docking assays for each T-cell epitope and its corresponding MHC molecule were conducted via the HPEPDOCK2.0 server [51, 52], and subsequent visualization was performed via PyMOL v3.1 software. Finally, the virtual screening tool LigPlot^+^ was used to assess the interactions between epitopes and HLA molecules [53].

### Design of the final vaccine sequence

The core components of the final vaccine include four key elements: adjuvants, HTL epitopes, CTL epitopes, and LBL epitopes. To achieve ordered assembly of epitopes with different functions and avoid spatial conformational interference, this study specifically employed three types of linkers for tandem connection: the AAA linker for CTL-CTL epitope ligation, whose short and rigid structure minimizes spatial steric hindrance, maintains the native conformation of epitopes, does not impair CTL activity, and is suitable for the independent recognition of CTL epitopes from target viruses [54]; the GGGGS linker for HTL-HTL epitope ligation, whose flexible and hydrophilic properties avoid epitope interference and maintain conformational flexibility to facilitate MHC-II binding and B-cell recognition [55]; the KK linker for LBL-LBL epitope ligation, whose positive charge enhances electrostatic interactions and T-cell cross-priming efficiency, and is compatible with MHC molecule binding to resist mixed infections [56].

To boost vaccine immunogenicity, two exogenous adjuvants were integrated into the core sequence: the tetanus toxin immunodominant epitope P2 (sequence: QYIKANSKFIGITEL) and the RS09 adjuvant (sequence: APPHALS). P2 exerts dual immunomodulatory effects: activating antigen-presenting cells (APCs) via regulating co-stimulatory molecule expression to improve antigen processing/presentation efficiency, and directing secretion of key cytokines (interleukins, interferons) to balance humoral and cellular immunity through immune cell differentiation/proliferation control [57, 58]. RS09, a widely used synthetic TLR-4 agonist, is recognized for robust immune activation and antibody production enhancement [59, 60].

To maintain structural integrity and independent functional activity of each domain, a rigid EAAAK linker bridged adjuvants and the HTL epitope module. Its stable α-helical conformation effectively separates adjuvant and epitope regions, avoiding spatial folding-induced functional interference [61]. Additionally, a 6 × His tag was added at the C-terminus as a specific marker for post-expression affinity purification, facilitating efficient separation and purification of the vaccine protein in subsequent processes.

### Prediction of the core biophysical and biochemical properties

This study employed the ProtParam server [62, 63] to compute the core physicochemical parameters, including the number of amino acid residues, theoretical isoelectric point, aliphatic index, instability index, theoretical in vitro half-life, and average hydrophilicity value. To validate the vaccine's antigenicity, this study utilized the VaxiJen 2.0 server for validation. To meet the safety screening requirements for allergenicity and toxicity, the AllerTOP v2.0 server and the ToxinPred v2.0 server were employed for predictive analysis of allergenicity and toxicity, respectively. Considering that cleavage of epitopes by signal peptidases may disrupt the structural integrity of the vaccine, we utilized the SignalP-6.0 server [64, 65] to predict potential signal peptides and their corresponding cleavage sites. Additionally, to exclude transmembrane structural elements that might affect epitope presentation efficiency, we predicted the transmembrane topology of the vaccine by the DeepTMHMM server. Furthermore, the predicted solubility of the vaccine construct was assessed by the Protein-Sol server [66, 67].

### Forecasting and optimization of secondary and tertiary structures

Leveraging position-specific scoring matrices, the PSIPRED4.0 server [68, 69] was used to predict the vaccine's secondary structure with high accuracy. We performed tertiary structure modeling of the vaccine using four servers concurrently: the Protenix server [70], the AlphaFold3 server [71], the AlphaFold2 server [72], and the Chai1 server [73]. Subsequently, we utilized the GalaxyWEB server [74, 75] to optimize the tertiary structure model of the vaccine, and selected the optimal model from the refined structures.

For in-depth validation, the optimized protein model was assessed using the SAVES v6.1 server [76, 77] to generate the ERRAT score. This score identifies error-prone regions in protein structures by analyzing atomic pairwise interactions, and models with an ERRAT score above 85 are recognized as high-quality. The Ramachandran plot was generated using the PROCHECK tool to evaluate the model's stereochemical quality. We considered the structure acceptable if over 90% of amino acid residues lay within the favored conformational regions. Additionally, the ProSA-web server [78, 79] was used to detect potential structural defects and to evaluate the overall model quality via the *Z*-score. A *Z*-score outside the typical range for native proteins of similar size may suggest potential conformational issues.

### Molecular docking

Toll-like receptors (TLRs) are key innate immune pattern recognition receptors that specifically recognize pathogen-associated molecular patterns. Upon recognition, they activate downstream NF-κB and MAPK signaling cascades, promoting dendritic cell (DC) maturation, enhancing antigen presentation, and facilitating helper T cell subset differentiation—ultimately bridging innate and adaptive immunity and mediating initial pathogen defense [80]. TLR2 and TLR4—among the most well-studied TLRs—recognize diverse pathogen components, with their activation efficiency directly shaping vaccine-induced immune response strength and type. They were therefore chosen as core targets to evaluate the vaccine’s immunostimulatory potential [81, 82].

For molecular docking, high-resolution crystal structures of TLR2 (PDB ID: 2Z7X) and TLR4 (PDB ID: 8WO1) were retrieved from the RCSB PDB database. The docking analysis was performed to evaluate the plausibility of vaccine–TLR interactions by exploring potential binding modes and interface complementarity, thereby providing a computational rationale for subsequent in vitro validation of immune activity. In parallel, HLA–vaccine docking was conducted because HLA-mediated epitope presentation is central to adaptive immunity and may influence the predicted population coverage and immunogenic potential of the designed construct.

Preliminary docking was conducted using the ClusPro 2.0 server [83, 84], which generates multiple conformations via cluster-based algorithms and selects optimal binding modes by energy scoring. Top-ranked conformations were submitted to the HADDOCK 2.4 server [85, 86] for complex optimization (default energy minimization parameters) to resolve atomic clashes and refine intermolecular interactions. The molecular basis of vaccine-TLR binding was analyzed using the PDBsum server [87, 88] on optimized complexes.

### Molecular dynamics simulation

To thoroughly validate the structural stability and binding reliability of the vaccine-TLRs complex in a dynamic environment, this study employed the Gromacs 2023.3 software [89, 90] to perform molecular dynamics (MD) simulations. The core objective was to evaluate the dynamic conformational changes and long-term stability of the post-docking complex under simulated physiological conditions; concurrently, we performed a systematic analysis of the simulation trajectory to explore the optimal equilibrium state of the system—defined by the lowest energy and most stable conformation.

For system construction, the vaccine-TLR complexes were parameterized with the Amber14SB_parmbsc1 force field (combining Amber14SB for proteins and parmbsc1 corrections), a widely used force field for accurate protein–protein complex structural and dynamic simulations [91]. Atomic coordinate and topology files were generated based on this force field. To mimic physiological charge neutrality and salt concentration, 0.145 mol/L NaCl was added to neutralize system charge and replicate the physiological ionic environment. Multiple rounds of energy minimization via the steepest descent method were conducted until convergence (maximum force ≤ 1000.0 kJ/mol/nm), eliminating atomic spatial clashes and local high-energy sites in the initial conformation.

In the equilibrium phase, sequential 500 ps NVT (isothermal-isochoric) and 500 ps NPT (isothermal-isobaric) simulations were performed: the NVT step stabilized the system at physiological temperature (310 K) using a modified Berendsen thermostat, while the NPT step adjusted pressure to 1 atm (Parinello-Rahman barostat) to stabilize system volume and density under physiological conditions. A 100 ns production MD simulation was then run under isothermal-isobaric conditions.

Throughout simulations, hydrogen bond lengths were constrained with the LINCS algorithm to maintain structural stability and calculation accuracy. Long-range electrostatic interactions were computed via the Particle-Mesh Ewald method, critical for accurately describing non-covalent vaccine-TLR interactions. Key structural metrics—including root mean square deviation (RMSD), root mean square fluctuation (RMSF), radius of gyration (Rg), and hydrogen bond count—were analyzed from the full 100 ns trajectory.

### MM-PBSA

To conduct a quantitative assessment of the strength of vaccine-receptor interactions, this study utilized the GMX_MMPBSA v1.62 analytical toolkit for computing and evaluating binding energy [92]. Specifically, we adopted the Molecular Mechanics/Poisson-Boltzmann Surface Area (MM-PBSA) approach, which provides an estimated binding free energy by accounting for contributions from molecular mechanical energy and solvation effects. During the computation process, to ensure the stability of the results, we extracted trajectory data covering the last 10 ns of the MD simulation as the analytical basis.

### Immune simulation

To gauge the potential immunogenic potency of the engineered vaccine, the present study performed in silico immune response simulation analysis by leveraging the C-ImmSim server [93, 94]. This tool recognizes epitopes through the Position Specific Scoring Matrix algorithm and is capable of simulating immune responses to epitopes in three mammalian tissues: the intestine, thymus, and lymph nodes. The total duration of the simulation was set to 350 days, corresponding to 1050 simulation steps (with each step representing 8 h). A three-dose regimen was adopted for the vaccination schedule: the first dose was administered on Day 1 (Step 1); the second and third doses, with a 4-week interval between them, were given on Day 28 (Step 84) and Day 56 (Step 168), respectively, with each dose containing 1000 vaccine units [95, 96].

### Population coverage prediction

Owing to their polymorphic nature, MHC molecules give rise to notable variations in the distribution patterns of HLA alleles across distinct geographical regions and diverse ethnic populations. To gauge the coverage rate of the designed vaccine among the global population, this study utilized the Population Coverage analysis tool [97, 98] provided by the IEDB database to estimate the vaccine's global coverage rate.

### Assembly of multi-epitope mRNA vaccine

First, the core target amino acid sequence was finalized. A tPA signal peptide (mediating vaccine protein secretion and enhancing antigen presentation [99]) was linked to its 5' end via an EAAAK rigid linker, while an MITD sequence (enabling targeted CTL epitope presentation [100]) was attached to the 3' end using another EAAAK linker. The rigid linkers ensure spatial separation of functional domains, allowing independent activity without conformational interference, and forming the complete target fusion amino acid sequence.

Subsequently, this fusion sequence was submitted to the Jcat server [101, 102] for reverse translation into an initial DNA sequence, followed by codon optimization to determine the vaccine’s core coding DNA. Next, a Kozak sequence (improving eukaryotic translation initiation [103]) was added to the 5' end of the optimized coding sequence; 5' UTR (maintaining mRNA structural stability) and 3' UTR (prolonging translation duration [104]) were linked to the two ends, respectively. A TAA stop codon was inserted upstream of the 3' UTR to ensure accurate translation termination.

Post regulatory element integration, BamHI (GGATCC) and XhoI (CTCGAG) restriction sites were inserted at the 5' and 3' ends of the DNA sequence, yielding the final vaccine template DNA with complete functional and regulatory elements. Finally, the template DNA was in vitro transcribed to generate mRNA, and its secondary structure was predicted via the RNAfold server [105, 106], providing core data for subsequent mRNA stability evaluation.

### Codon optimization verification and in silico cloning

To further ensure the expression efficiency of the vaccine in the host, this study performed secondary targeted optimization on the core coding DNA sequence of the vaccine using the Jcat server, with the codon preference of Escherichia coli K12 strain as the benchmark. Simultaneously, the Codon Adaptation Index (CAI) and GC content of the optimized sequence were analyzed—CAI is a core indicator for measuring the expression efficiency of prokaryotic genes, while GC content directly affects the transcriptional stability and translational efficiency of mRNA.

After the completion of optimization verification, the "template DNA sequence containing regulatory elements + restriction sites" constructed in Sect. "Assembly of Multi-Epitope mRNA vaccine" was used as the target fragment. In silico cloning was performed via the GenSmart platform, and the fragment was inserted into the pET-28a (+) vector [107].

Detailed information on the comprehensive bioinformatics parameters employed throughout the entire development process of the trivalent multi-epitope mRNA vaccine, including target protein screening, epitope prediction, vaccine design, molecular docking, molecular dynamics simulation, and immune/coverage evaluation, is summarized in Supplementary Table S1. Additionally, Supplementary Table S6 provides a complete list of the bioinformatics software and online servers utilized in this study, along with their corresponding versions, affiliated institutions, and countries of origin.

## Results

### Screening of target proteins, dominant subtypes and representative sequences

Based on the multi-dimensional screening system established in Sect. "Initial sequence retrieval and preliminary protein screening", this study conducted progressive stepwise screening on the initial protein libraries of Norovirus, Rotavirus, and Adenovirus 40/41, and finally identified 6 target proteins that meet the core criteria. The core screening criteria include: antigenicity score > 0.4, no toxic domains, non-allergenic, number of transmembrane helices < 1, and subcellular localization in the viral capsid, extracellular space, or cell membrane surface. The information of target proteins classified by virus type is as follows: Norovirus: major capsid protein VP1; Rotavirus: adhesion protein VP4, core protein VP6, capsid protein VP7; Adenovirus 40/41: Hexon protein, short fiber protein.

Given that all the aforementioned viruses have multiple epidemic subtypes, this study screened the dominant epidemic subtypes of each virus based on the latest epidemiological data to improve the broad-spectrum coverage of the vaccine. The specific subtypes are as follows: Norovirus VP1 covers subtypes GI.3, GI.6, GII.2, GII.4, and GII.6; Rotavirus VP4 covers genotypes P[4], P[6], and P[8], VP6 is restricted to group A (predominant clinical group), and VP7 covers genotypes G1, G2, G3, G9, and G12; both Hexon protein and short fiber protein of Adenovirus 40/41 cover the two pathogenic subtypes (type 40 and type 41).

To clarify the representative sequences of each subtype for supporting subsequent epitope screening, this study performed phylogenetic analysis on all quality-controlled full-length sequences of each subtype, and finally determined the representative reference sequences of each subtype (with GenBank accession numbers listed below): Norovirus VP1 subtypes: GI.3 (WYW48828.1), GI.6 (AFH88383.1), GII.2 (AUD39963.1), GII.4 (ANP93502.1), GII.6 (ANY58562.1); Rotavirus VP4 genotypes: P[4] (XDL44633.1), P[6] (BBD06625.1), P[8] (XQU58909.1), VP6 (AIN41107.2), VP7 genotypes: G1 (ADC32676.1), G2 (WDU50760.1), G3 (YBQ72798.1), G9 (ADT62510.1), G12 (BAI49627.1); Adenovirus 40/41: Hexon protein (WFP21729.1), short fiber protein (WOR09025.1). Since the number of available full-length sequences of Hexon and short fiber protein from adenovirus 40/41 is limited, making the construction of separate phylogenetic trees for each subtype impractical, and the sequence conservation of these proteins between the two subtypes is high (> 95%), the same representative sequence was therefore used to cover both subtypes.

**Fig. 1:**
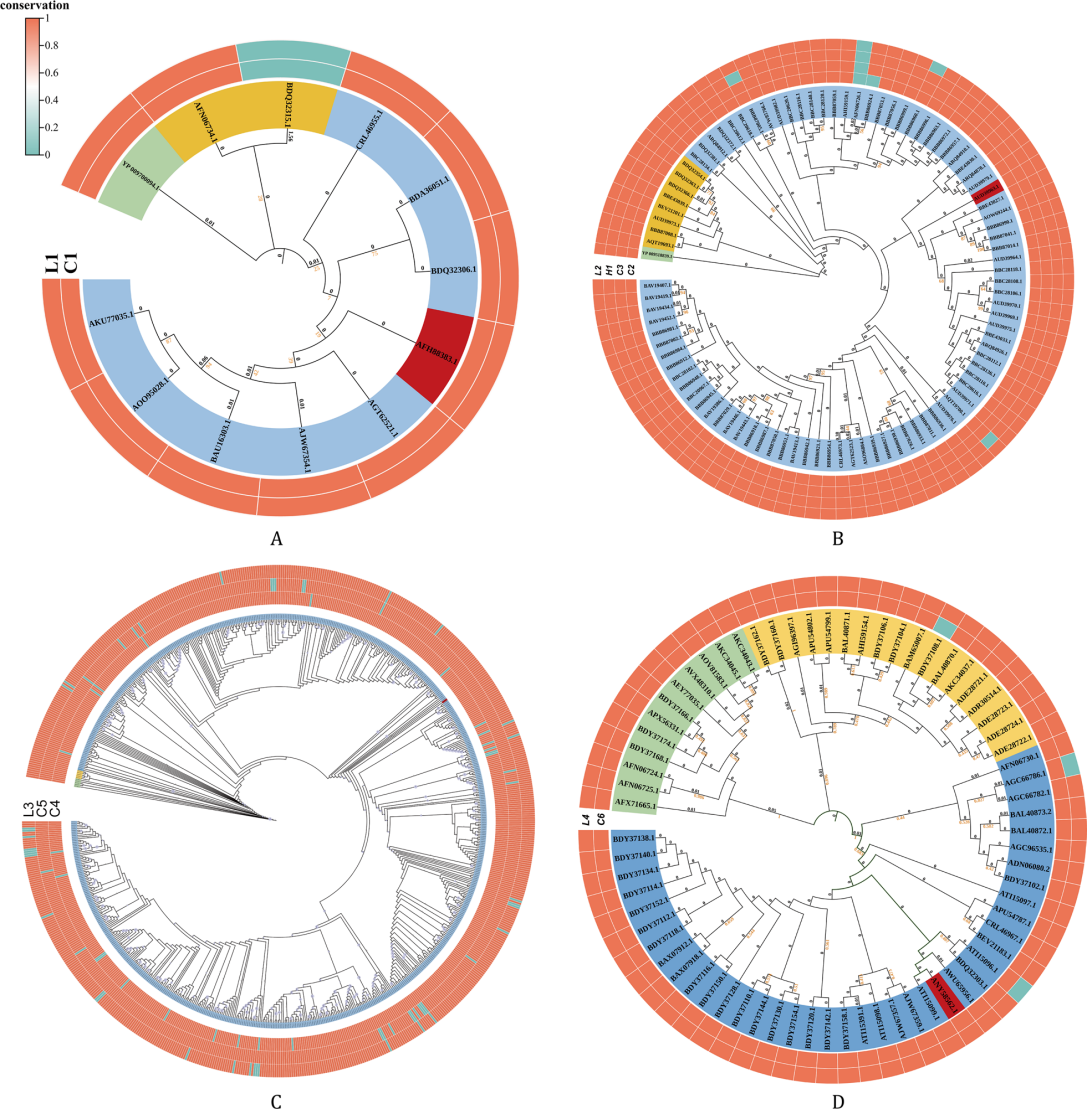
Phylogenetic tree and epitope conservation analysis of VP1 proteins from different norovirus subtypes (Inner circle: phylogenetic tree, with red sequences representing the target sequences selected for epitope screening; Outer circle: distribution of epitope conservation). (**A**) GI.6; (**B**) GII.2; (**C**) GII.4; (**D**) GII.6

All the aforementioned representative strains meet the criterion that the sum of their patristic distances to all other sequences in the phylogenetic tree is minimized, and the total evolutionary distance between this reference sequence and all sequences of the corresponding subtype is also minimized. The phylogenetic analysis results of some Norovirus subtypes are shown in Fig. 1 (it should be noted that the phylogenetic tree of Norovirus subtype GI.3 was not included in Fig. 1, as no qualified candidate epitopes were obtained for this subtype in the subsequent epitope screening); the phylogenetic analysis results of different Rotavirus subtypes are shown in Figs. S1-S2; the relevant analysis results of different Adenovirus subtypes are shown in Fig. S3.

### Epitope identification, secondary validation and HLA binding analysis

Taking the representative sequences of each viral subtype as target molecules, this study finally identified 16 CTL epitopes (Table 1), 5 HTL epitopes (Table 2), and 17 LBL epitopes (Table 3) through multiple rounds of epitope prediction, screening, and functional validation. These epitopes serve as the core functional components for the construction of multi-epitope vaccines. All candidate epitopes meet strict screening thresholds, including: strong binding potential to target molecules, antigenicity score > 0.4, no potential toxicity or allergenicity, cross-population MHC-binding capacity, excellent immunogenicity score, and MHC presentation score > 0. Among these, HTL epitopes are additionally required to possess the biological function of inducing the secretion of key cytokines (IFN-γ, IL-4) beyond meeting the aforementioned basic criteria. Furthermore, regarding the characteristic of rotavirus VP4 protein—following entry into the host intestine, it undergoes protease cleavage into two functional domains, VP5 and VP8—this study performed additional validation on the candidate epitopes screened from the VP4 protein. By comparing the start positions of the epitopes in the full-length VP4 sequence, we ensured that all epitopes are localized within the VP5 or VP8 functional domains formed after cleavage, rather than in the excised cleavage region, thereby supporting the immunological accessibility of the epitopes during viral infection. The detailed screening process and data of epitope screening can be found in Supplementary Material 2.

**Table 1:** Screen of cytotoxic T lymphocyte epitopes

Protein	Subtype	Sequence	Start	EL percentile	BA IC50	Processing total score	Antigenicity	Toxicity	Allergenicity	Allele	Conservation
VP1	GI.6	FVFVSWVSR	496	0.06	17.6	0.57968	1.4862	–	–	HLA-A*33:01	91.67% (11/12)
	GII.2	MSEVALVRY	471	0.01	18.53	1.352837	0.4391	–	–	HLA-A*01:01	97.87% (92/94)
		KMRIVAMLY	175	0.04	11.1	1.558646	0.8190	–	–	HLA-A*30:02	98.94% (93/94)
	GII.4	IFAAVPPNF	120	0.01	13.52	1.413224	0.5720	–	–	HLA-A*23:01	96.99% (709/731)
		SQVTMFPHI	136	0.14	9.96	0.371862	0.6173	–	–	HLA-A*02:06	96.72% (707/731)
	GII.6	LPILTLGEL	228	0.12	37.87	0.195058	0.8355	–	–	HLA-B*07:02	98.57% (69/70)
VP4	P[4]	IMNGGAVSL	395	0.09	20.48	0.712337	0.6138	–	–	HLA-A*02:03	97.67% (168/172)
	P[6]	YEIAGRFSL	461	0.01	4.65	1.00681	0.7587	–	–	HLA-B*40:01	94.34% (50/53)
	P[8]	ILRTRTVNL	438	0.01	18.83	0.951492	1.1562	–	–	HLA-B*08:01	98.99% (393/397)
		VMNGGAVSL	395	0.1	31.94	0.536413	0.6197	–	–	HLA-A*02:03	96.47% (383/397)
VP6	A	RVFTVASIR	384	0.03	8.66	1.058141	0.6169	–	–	HLA-A*31:01	97.20% (936/963)
											96.67% (174/180)
VP7	G1\G3\G9	NPMDITLYY	166	0.01	3.28	2.028714	1.4099	–	–	HLA-B*35:01	92.68% (228/246)
											98.25% (225/229)
	G2	TVVDYINQI	298	0.01	14.84	0.27904	0.4108	–	–	HLA-A*68:02	91.15% (103/113)
hexon	41\41	SYKDRMYSF	760	0.01	29.55	1.095804	1.3403	–	–	HLA-A*24:02	100.00% (21/21)
		RSMLLGNGR	520	0.06	7.59	0.949477	1.0261	–	–	HLA-A*31:01	100.00% (21/21)
		TELSYQLML	343	0.05	23.25	0.601819	1.0004	–	–	HLA-B*40:01	100.00% (21/21)

**Table 2:** Screen of helper T llymphocyte epitopes

Protein	Subtype	Sequence	EL percentile	BA IC50	Antigenicity	Toxicity	Allergenicity	INF-γ	IL-4	Allele	Conservation
VP1	GII.2	AKLHRAGFMTVSSNT	1.7	37.37	0.4472	–	–	+	+	HLA-DPA1*01:03/DPB1*02:01	96.81% (91/94)
VP4	P[4]/P[6]/P[8]	IDFKTLKNLNDNYGI	0.08	21.41	1.1012	–	–	+	+	HLA-DRB1*04:05	100.00% (172/172), 96.23% (51/53), 98.24% (390/397)
	P[4]/P[8]	WKEMQYNRDIIIRFK	0.58	11.35	0.4191	–	–	+	+	HLA-DRB3*01:01	97.09% (167/172), 97.23% (386/397)
hexon	40/41	EWNFRKDVNMILQSS	0.17	12.66	0.5836	–	–	+	+	HLA-DRB3*01:01	100.00% (21/21)
		GSYTYEWNFRKDVNM	0.59	46.35	0.6351	–	–	+	+	HLA-DRB5*01:01	100.00% (21/21)

**Table 3:** Screen of linear B lymphocyte epitopes

Protein	Subtype	Sequence	Score	Antigenicity	Toxicity	Allergenicity	Conservation
VP1	GI.6	HNSSQPQPTMRLVAML	0.84	0.5028	–	–	91.67% (11/12)
	GII.2	GPELNPYLAHLARMYN	0.73	0.5017	–	–	97.87% (92/94)
	GII.4	GPDLNPYLSHLARMYN	0.69	0.4726	–	–	95.90% (701/731)
	GII.6	PGEMLLNLELGPELNP	0.89	1.3077	–	–	97.14% (68/70)
VP4	P[4]/P[8]	DITISKTSLWKEMQYN	0.89	0.5334	–	–	95.35% (164/172), 91.94% (365/397)
	P[4]/P[6]/P[8]	DFVSLNSLRFRFSLTV	0.79	1.3029	–	–	100.00% (172/172), 98.11% (52/53), 98.49% (391/397)
		IDFKTLKNLNDNYGIT	0.53	1.1012	–	–	99.42% (171/172), 96.23% (51/53), 98.24% (390/397)
VP6	A	NNVEVEFLLNGQIINT	0.53	0.7315	–	–	91.69% (883/963)
VP7	G1	PMDITLYYYQQSGESN	0.73	0.8518	–	–	90.56% (163/180)
	G2	LMRYDNTSELDASELA	0.73	0.4843	–	–	95.58% (108/113)
		EKLVITDVVNGVNHKI	0.83	0.4437	–	–	90.27% (102/113)
	G12	PTTIPQTERMMRINWK	0.81	1.1025	–	–	98.51% (66/67)
hexon	40/41	DRSQRLTLRFVPVDRE	0.79	1.3292	–	–	100.00% (21/21)
		PNYIGFRDNFIGLMYY	0.65	1.3120	–	–	100.00% (21/21)
		NHHRNAGLRYRSMLLG	0.77	1.1061	–	–	100.00% (21/21)
Short fiber protein	40/41	TFMPNSTVYPRNKTAD	0.88	0.8850	–	–	92.31% (24/26)
		LIQISPNITFSVVYNE	0.87	0.9945	–	–	92.31% (24/26)

Conservation analysis revealed that all candidate epitopes exhibit excellent conservation in their corresponding viral subtypes, with conservation values all > 90% (Tables 1, 2 and 3). To intuitively present the conservation characteristics of the epitopes, this study used a circular heatmap to label the conservation results of each epitope on the outer ring of the phylogenetic tree, enabling the visual correlation between conservation data and evolutionary relationships (Fig. 1, Figs. S1-S3).

Prediction results from MixMHCpred v3.0 and MixMHC2pred v2.0 showed that CTL and HTL epitopes exhibited a certain degree of functional hierarchy. Among the 16 CTL epitopes, 9 core epitopes (e.g., NPMDITLYY, SYKDRMYSF) all met the screening criterion of "%Rank_bestAllele (percentile rank of best allele) < 0.1%", could target globally high-frequency HLA-I alleles such as A*01:01, A*02:06, and B*35:01, with a theoretical population coverage rate reaching 78.2%. The remaining 7 auxiliary epitopes (e.g., FVFVSWVSR, LPILTLGEL) had %Rank_bestAllele values ranging from 0.12% to 0.19%, which could supplement the coverage of populations carrying minor alleles like A*68:01 and B*15:01, and may help reduce the risk of vaccine immune escape. Among the 5 candidate HTL epitopes, 4 high-quality epitopes were identified. Among them, 3 core high-quality epitopes (e.g., IDFKTLKNLNDNYGI, best binding allele: DRB1*04:05, %Rank = 0.157%) could bind to high-frequency alleles of the DR subfamily (SubSpec_best = 1), effectively activate CD4⁺ T cells, and assist in the activation of CD8⁺ T cells and B cell-derived antibody production. There was also 1 auxiliary high-quality epitope, GSYTYEWNFRKDVNM (best binding allele: DRB5*01:01, %Rank = 0.789%), which may help improve the vaccine coverage in DRB5*01:01-positive populations.

The BLASTp results showed that all 38 candidate epitopes fully passed both conservancy and safety verification: in terms of conservancy, the E-values for the alignment between all epitopes and the corresponding subtype sequences of the target pathogen ranged from 1.00E-15 to 4.00E-04, with both Query Coverage and Identity exceeding 95%, indicating no significant variation of the epitopes among circulating strains, while regarding safety, the human-matched E-values ranged from 11 to 139 (all > 0.05) and no significant homology with human proteins was detected, minimizing the risk of autoimmune reactions. In summary, all 38 epitopes exhibit potential "high conservation, high safety, and broad spectrum" characteristics, offering potential candidate targets for subsequent vaccine construction (Supplementary Tables S2-S4).

**Fig. 2:**
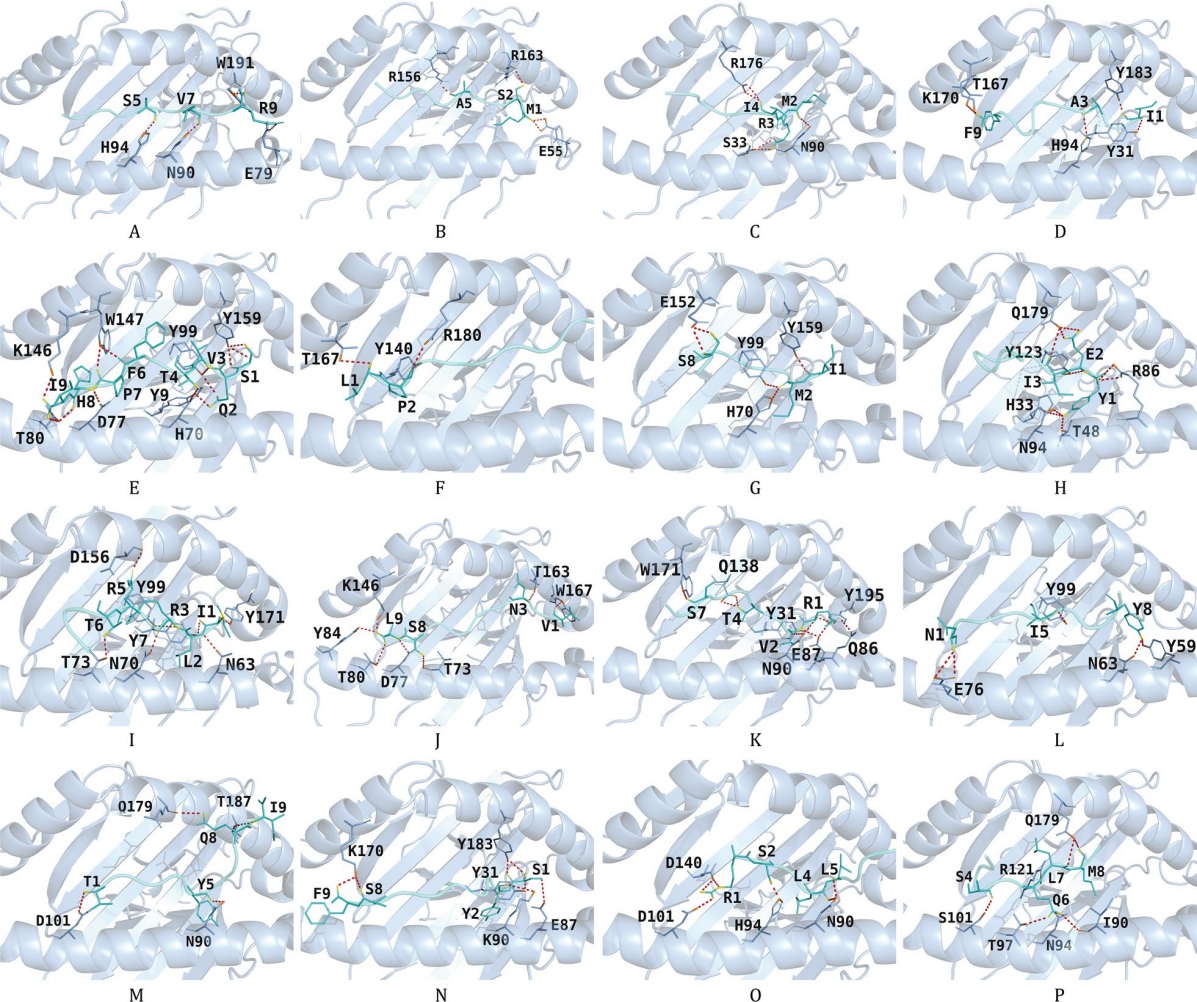
The schematic diagram of docking between each of the 16 CTL epitopes and HLA molecules

To further verify the immunological activity of T-cell epitopes, this study performed molecular docking experiments between epitopes and HLA molecules. This docking analysis not only enables the quantitative assessment of the binding affinity and conformational compatibility between epitopes and MHC molecules but also deciphers key interaction patterns such as hydrogen bonds and hydrophobic interactions between them, thereby systematically verifying whether the selected epitopes can be effectively recognized, bound, and presented by immune cells. The results showed that all T-cell epitopes formed sufficient hydrogen bonds with their corresponding HLA molecules, suggesting they possess effective binding capacity (Fig. 2, Fig. S4). In addition, visualization analysis of epitope-HLA interactions was conducted via the LigPlot^+^ tool, which further indicated that all T-cell epitopes had adequate interactions with their corresponding HLA molecules (Figs. S5-S6).

### Vaccine construction and characteristic assessment

**Fig. 3:**
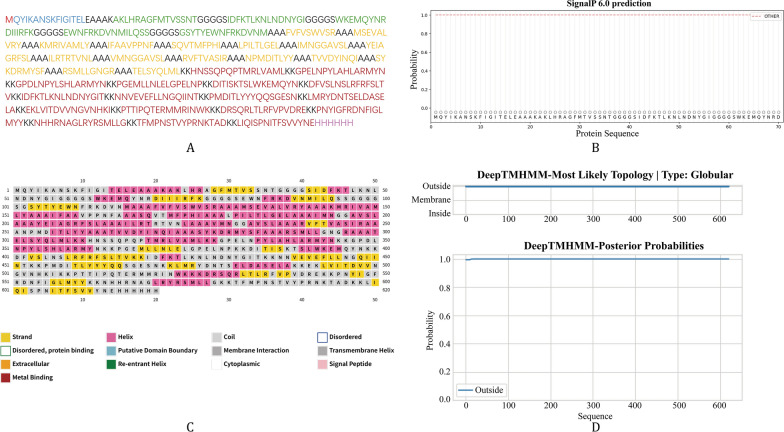
Vaccine constructed with Tetanus Toxin P2 adjuvant: analysis of sequence, secondary structure, signal peptide, and transmembrane helices. (**A**) Amino acid sequence (color coding: blue represents adjuvant; black represents linkers; green represents HTL epitopes; yellow represents CTL epitopes; red represents LBL epitopes; purple represents 6 × His tag); (**B**) Signal peptide; (**C**) Secondary structure; (**D**) Transmembrane helices. HTL: Helper T lymphocyte; CTL: Cytotoxic T lymphocyte; LBL: Linear B lymphocyte

In this study, two adjuvants, tetanus toxin P2 and RS09, were selected to construct two candidate vaccine sequences. Both candidates shared an identical core design, comprising an adjuvant, 16 CTL epitopes, 5 HTL epitopes, 17 LBL epitopes, appropriate linkers, and a 6 × His tag (Fig. 3, Fig. S7).

For the candidate incorporating tetanus toxin P2, the VaxiJen 2.0 server predicted an antigenicity score of 0.7803. Analyses using ToxinPred 2.0 and AllerTop 2.0 server indicated it was likely non-toxic and non-allergenic. The candidate with RS09 adjuvant had a slightly lower antigenicity score (0.7676) and was also predicted to be non-toxic and non-allergenic by the corresponding tools. However, DeepTMHMM analysis indicated the presence of signal peptides in the RS09 construct, which is typically undesirable for a vaccine designed for cytosolic expression. Furthermore, its solubility coefficient predicted by the Protein-Sol server was 0.447, below the tool’s threshold of 0.45. Consequently, the RS09 candidate was excluded from further development. In summary, the vaccine sequence containing the tetanus toxin P2 adjuvant was selected as the lead candidate for subsequent investigations.

The final vaccine construct comprises 620 amino acids, with a molecular mass of 69.28 kDa. Its predicted isoelectric point is 10.31, suggesting a strong positive charge at physiological pH. In silico half-life predictions indicate stability of approximately 30 h in mammalian reticulocytes, > 20 h in yeast, and > 10 h in Escherichia coli. The instability index is 28.12, classifying it as a stable protein. The aliphatic index of 83.32 suggests potential for good thermal stability, and the grand average of hydropathicity (GRAVY) of -0.268 indicates a hydrophilic character. The Protein-Sol server predicted a solubility coefficient of 0.462, indicating favorable solubility. Analyses using SignalP-6.0 and DeepTMHMM confirmed the absence of signal peptides and transmembrane helices (Fig. 3B, D).

### Prediction and validation of secondary and tertiary structures

**Fig. 4:**
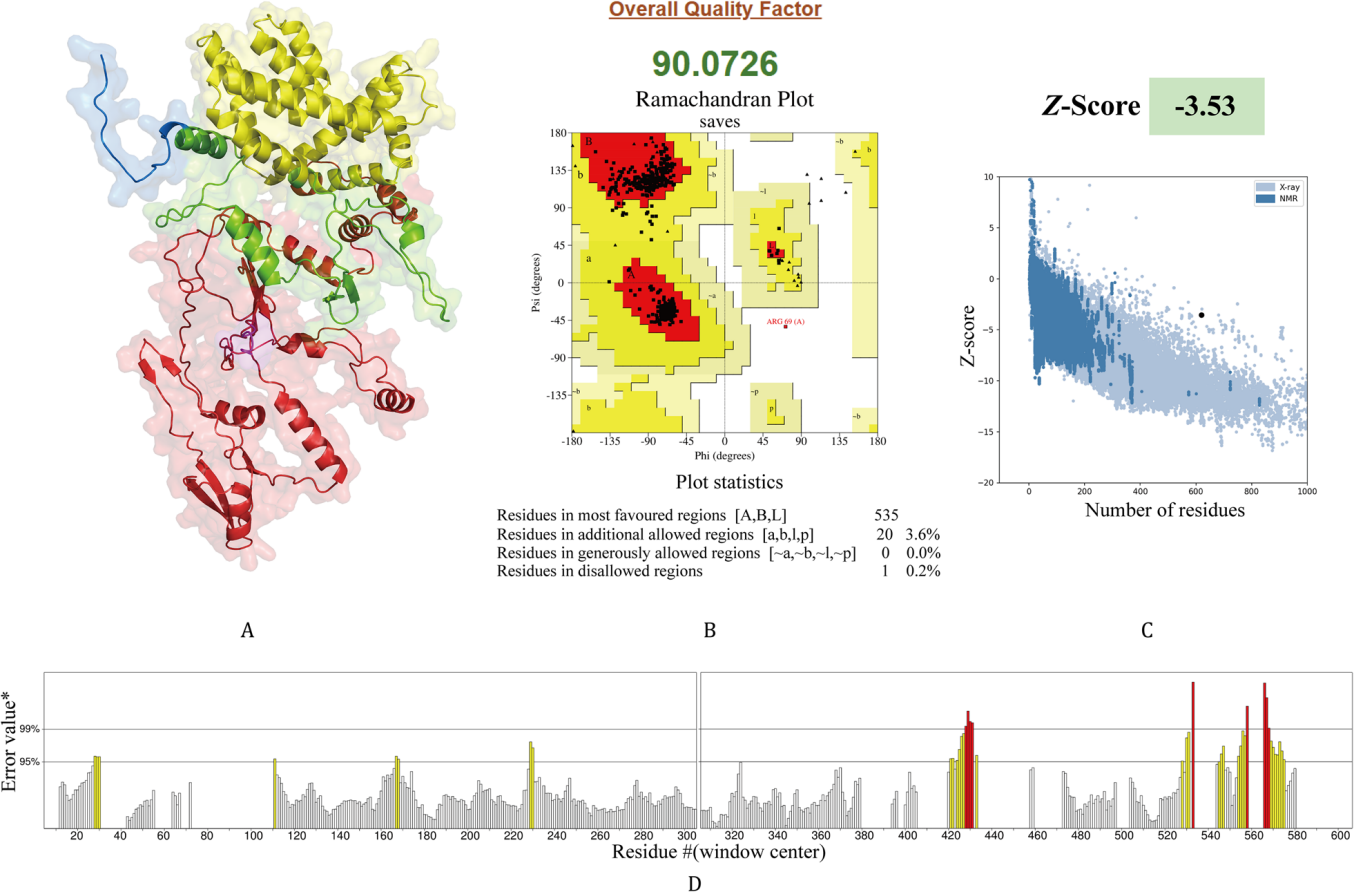
Tertiary structure and quality assessment. (**A**) Tertiary structure diagram (color coding: blue represents adjuvant; green represents HTL epitopes; yellow represents CTL epitopes; red represents LBL epitopes; purple represents 6 × His tag); (**B**) Ramachandran plot; (**C**) *Z*-score and energy plot; (**D**) Error value of vaccine residue. HTL: Helper T lymphocyte; CTL: Cytotoxic T lymphocyte; LBL: Linear B lymphocyte

As predicted by PSIPRED 4.0, the secondary structure of the vaccine is composed of 42.09% coils, 40.97% helices, and 16.94% strands (Fig. 3C). For tertiary structure analysis, initial 3D models of the vaccine were first constructed via four servers, namely ProteniX, Chai1, AlphaFold3, and AlphaFold2, followed by uniform structural optimization of all initial models using the GalaxyWEB server. To systematically evaluate the reliability of the optimized models, this study conducted a comprehensive quality assessment using three professional tools: ERRAT, PROCHECK, and ProSA-web. Based on the aforementioned assessment results, the model constructed by the AlphaFold3 server was ultimately selected as the final tertiary structure of the vaccine due to its superior quality among all candidate models. Specifically, this model achieved an ERRAT score of 90.0726 and a Z-score of − 3.53 via ProSA-web analysis. Ramachandran plot analysis by PROCHECK revealed that 96.2% of the amino acid residues were located in the most favored regions, and 3.6% were in the additionally allowed regions (Fig. 4).

### Molecular docking

To investigate the binding affinity and interaction mechanism between the vaccine and TLRs, as well as HLA molecules, this study first performed molecular docking analysis using the ClusPro 2.0 server, followed by structural optimization of the preliminary docking results via the HADDOCK server. Energy analysis results showed that the cluster energy and lowest binding energy of each complex were as follows: the vaccine-TLR2 complex had a cluster energy of − 1098 kcal/mol and a lowest binding energy of − 1385.2 kcal/mol; the vaccine-TLR4 complex had a cluster energy of − 956.3 kcal/mol and a lowest binding energy of − 1029.5 kcal/mol; the vaccine-HLA-A*02:01 complex had a cluster energy of − 1180.6 kcal/mol and a lowest binding energy of − 1342.6 kcal/mol; the vaccine-HLA-DRB1*01:01 complex had a cluster energy of − 1128.4 kcal/mol and a lowest binding energy of − 1187.9 kcal/mol. After optimization by the HADDOCK server, the HADDOCK scores of the complexes were -335.5 kcal/mol (TLR2), − 394.3 kcal/mol (TLR4), − 352.4 kcal/mol (HLA-A*02:01), and − 345.8 kcal/mol (HLA-DRB1*01:01), respectively.

**Fig. 5:**
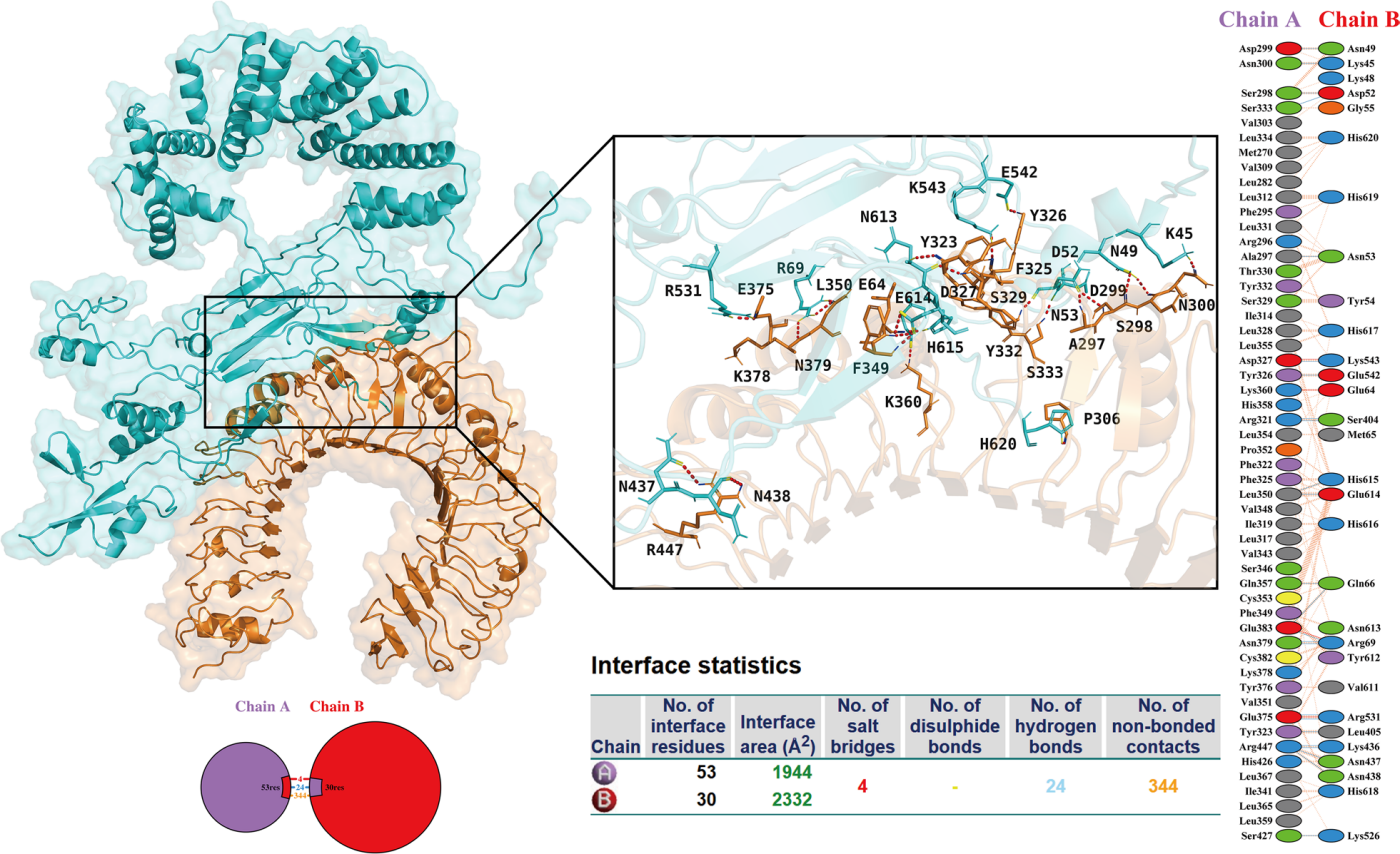
Docking results of the vaccine-TLR2 complex: the docking outcome is displayed on the left panel, and analysis regarding intra-complex binding interactions is presented on the right panel. This accompanying table provides detailed information on salt bridges, disulfide bonds, hydrogen bonds, and non-hydrogen bonding interactions. TLRs: Toll-like receptors

**Fig. 6:**
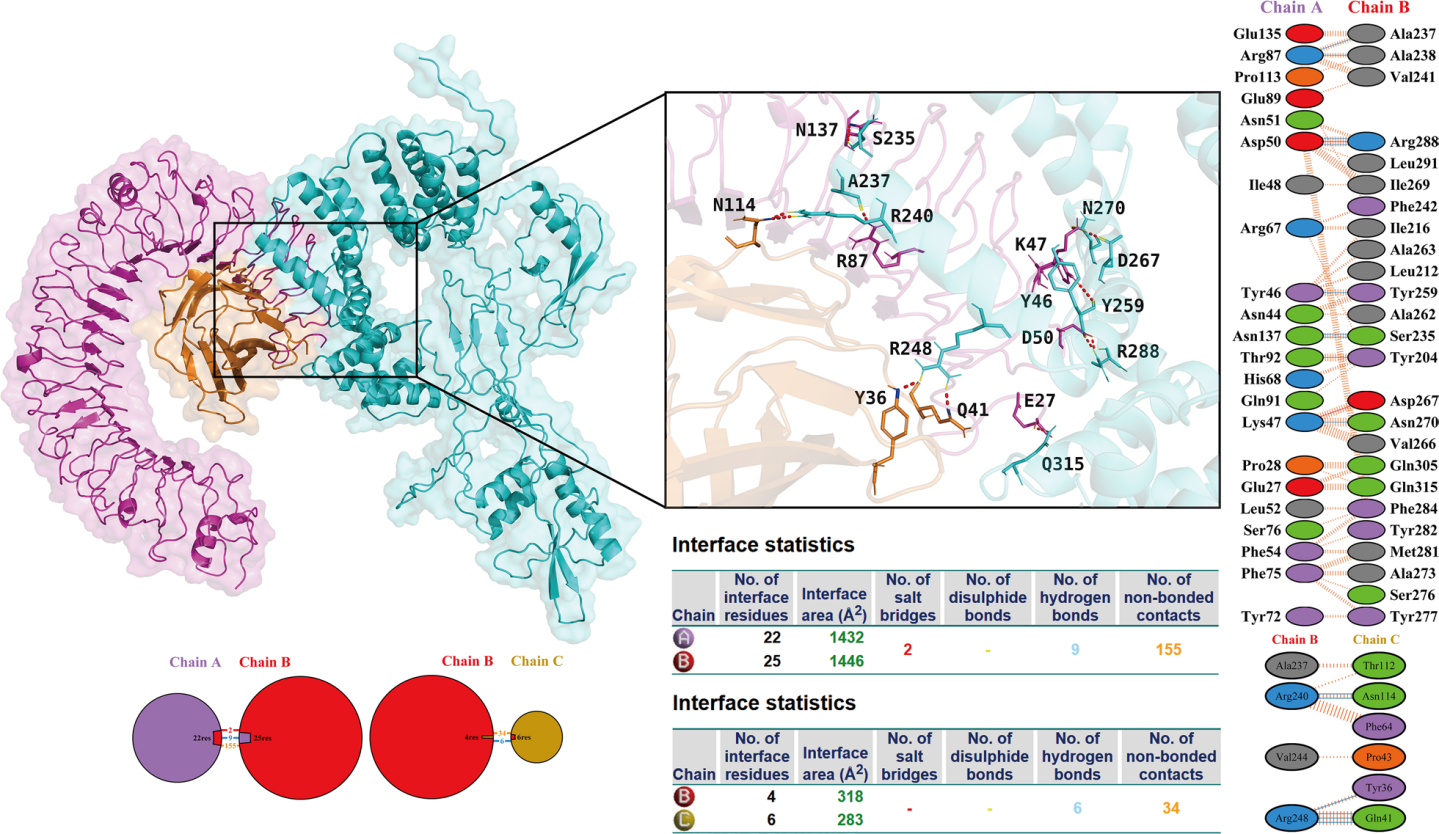
Docking results of the vaccine-TLR4 complex. TLRs: Toll-like receptors

To systematically characterize the binding interface interaction features of the complexes formed between the vaccine and TLRs/HLA molecules, this study conducted a dedicated assessment using the PDBsum server. This analysis further indicated that multiple hydrogen bonds were formed at the protein–protein binding interface, and these hydrogen bonds play a crucial role in maintaining the structural stability of the complexes. Specifically, the vaccine and TLR2 formed 24 hydrogen bonds at the binding interface (Fig. 5), 15 hydrogen bonds with TLR4 (Fig. 6), 37 hydrogen bonds with HLA-A*02:01 (Fig. S8), and 17 hydrogen bonds with HLA-DRB1*01:01 (Fig. S9). Additionally, information on the key amino acid residues mediating hydrogen bond formation between the vaccine and TLRs is summarized in Table 4.

**Table 4:** Key amino acid residues mediating the formation of hydrogen bonds

Receptor type	Key amino acid residues of vaccine	Key amino acid residues of receptor
Toll-like receptors 2	Asn49	Asp299
	Lys45	Asn300
	Asp52	Ser298, Ser333
	Asn53	Ala297
	Lys543	Asp327
	Glu542	Tyr326
	Ser404	Arg321*
	Leu350	His615
	Glu614	Leu350
	Gln66	Gln357, Glu383
	Arg69	Glu383, Asn379*
	Arg531	Glu375*
	Leu405	Tyr323
	Lys436	Arg447*
	Asn437	Arg447
	Asn438	Arg447
	Lys526	Ser427
Toll-like receptors 4	Ala237	Arg87
	Ala238	Arg87
	Arg288	Asp50*
	Tyr259	Tyr46
	Ser235	Asn137*
	Tyr204	Thr92
	Asn270	Lys47
	Arg240	Asn114
	Arg248	Tyr36, Gln41**

*indicates that this amino acid forms two hydrogen bonds with the corresponding amino acid, and ** indicates that this amino acid forms three hydrogen bonds with the corresponding amino acid

To further confirm the interaction details, this study utilized the LigPlot^+^ tool for visual analysis of intermolecular interactions in the vaccine-TLR/HLA complexes. The results further verified the presence of abundant hydrogen bonds and hydrophobic interactions in these complexes, which is consistent with the conclusions of the aforementioned molecular docking analysis and energy analysis (Figs. S10, S11).

### MD simulations

**Fig. 7:**
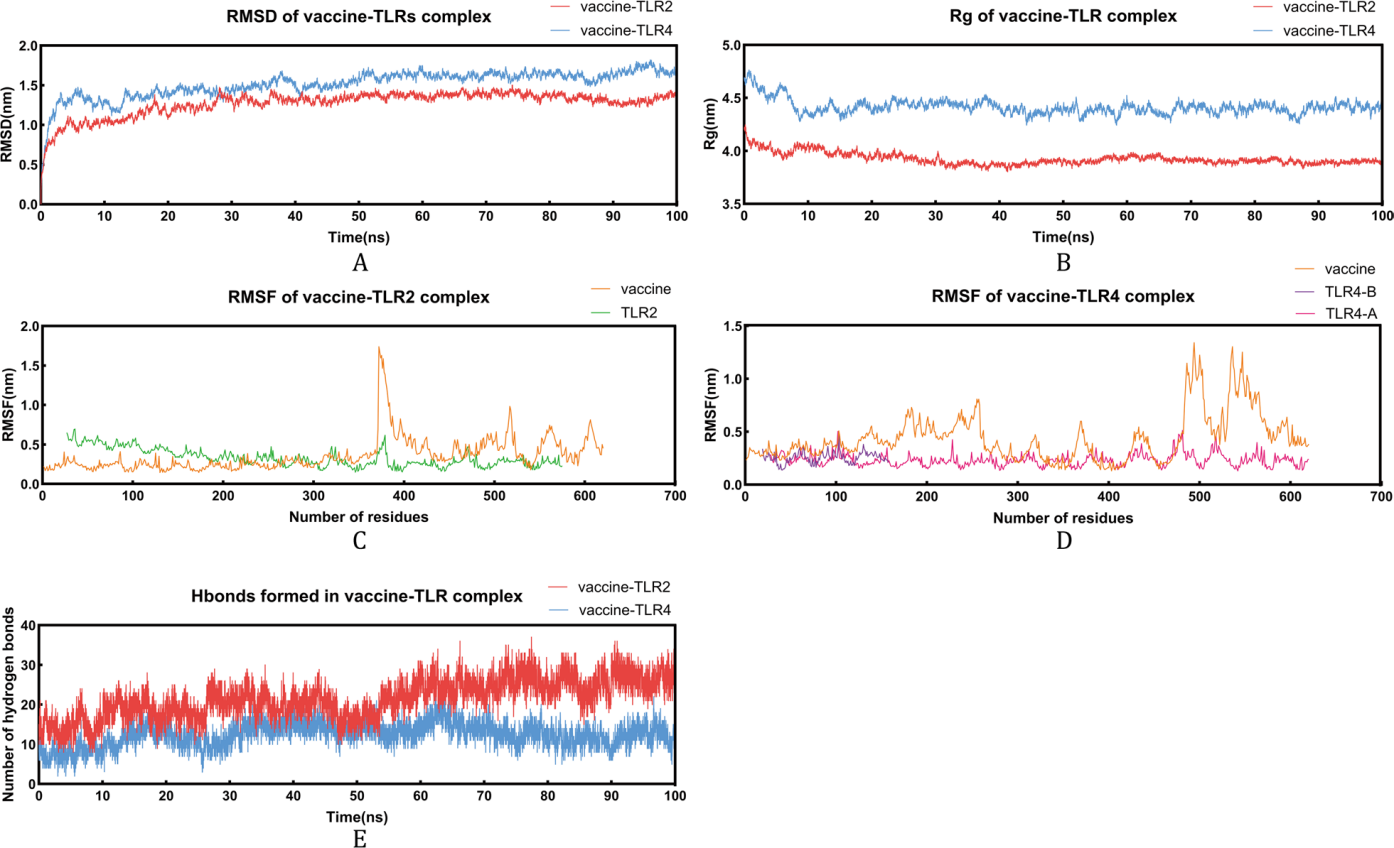
MD simulations of vaccine-TLR binding complexes. (**A**) RMSD profiles of vaccine-TLR binding complexes; (**B**) Rg profiles for vaccine-TLR binding complexes; (**C**, **D**) RMSF analyses of vaccine-TLR binding complexes; (**E**) Statistics on the number of hydrogen bonds. MD: molecular dynamics; TLRs: Toll-like receptors; RMSD: Root mean square deviation; RMSF: Root mean square fluctuation

This study used Gromacs v2023.3 to perform 100 ns MD trajectory analysis on vaccine-TLR (TLR2, TLR4) complexes to investigate dynamic structural changes (Fig. 7). RMSD, a quantitative measure of atomic-level conformational differences from the initial state, showed the vaccine-TLR2 complex RMSD rose irregularly in the first 30 ns, equilibrated at 30 ns, and remained stable until 100 ns; the vaccine-TLR4 complex RMSD increased irregularly in the first 40 ns, then stabilized for the rest of the simulation. Average RMSD values were 1.272 ± 0.155 nm (vaccine-TLR2) and 1.515 ± 0.172 nm (vaccine-TLR4), confirming good stability for both complexes (Fig. 7A).

Rg, a core indicator of structural compactness (quantifying the root-mean-square distance from a molecule’s atomic centroid), was analyzed: the vaccine-TLR2 complex Rg declined slowly and irregularly in the first 30 ns before stabilizing, while the vaccine-TLR4 complex Rg showed a similar irregular downward trend in the first 20 ns, followed by stability. Average Rg values were 3.926 ± 0.055 nm (vaccine-TLR2) and 4.415 ± 0.069 nm (vaccine-TLR4), indicating both complexes maintained compact structures (Fig. 7B). RMSF analysis revealed high flexibility in residues 370–440 and 480–620 of the vaccine-TLR2 complex, and residues 120–280 and 490–620 of the vaccine-TLR4 complex (Fig. 7C, D).

Intermolecular hydrogen bond count, a key measure of binding strength, was assessed: vaccine-TLR2 hydrogen bonds increased gradually in the first 50 ns and stabilized thereafter, rising from an initial 15 to a peak of 37 (average 21.422); vaccine-TLR4 hydrogen bonds increased in the first 20 ns, decreased slightly between 20 and 30 ns, then stabilized, rising from an initial 11 to a maximum of 23 (average 12.421) (Fig. 7E).

GMX_MMPBSA v1.62 was used to estimate MM/PBSA binding free energies, yielding average ΔG_total values of − 226.61 kcal·mol⁻^1^ for the vaccine–TLR2 complex and − 147.99 kcal·mol^− 1^ for the vaccine–TLR4 complex. The negative ΔG_total values suggest energetically favorable interactions and are consistent with stable associations observed during the simulations (Table 5).

**Table 5:** Molecular mechanics/Poisson-Boltzmann surface area binding free energy calculations for vaccine-TLR2 and TLR4 complexes. TLRs: Toll-like receptors; *SD*: Standard deviation

Energy component	ΔVDWAALS: Average (*SD*)	ΔEEL: Average (*SD*)	ΔEPB: Average (*SD*)	ΔENPOLAR: Average (*SD*)	ΔGGAS: Average (*SD*)	ΔGSOLV: Average (*SD*)	ΔTOTAL: Average (*SD*)
Vaccine-TLR2	− 352.71 (10.76)	− 2552.86 (117.51)	2716.79 (114.21)	− 37.82 (1.17)	− 2905.58 (121.02)	2678.96 (113.61)	− 226.61 (24.42)
Vaccine-TLR4	− 178.35 (11.10)	− 3236.72 (84.71)	3288.76 (83.26)	− 21.68 (1.00)	− 3415.07 (86.12)	3267.08 (83.09)	− 147.99 (12.36)

### Immunization simulation

**Fig. 8:**
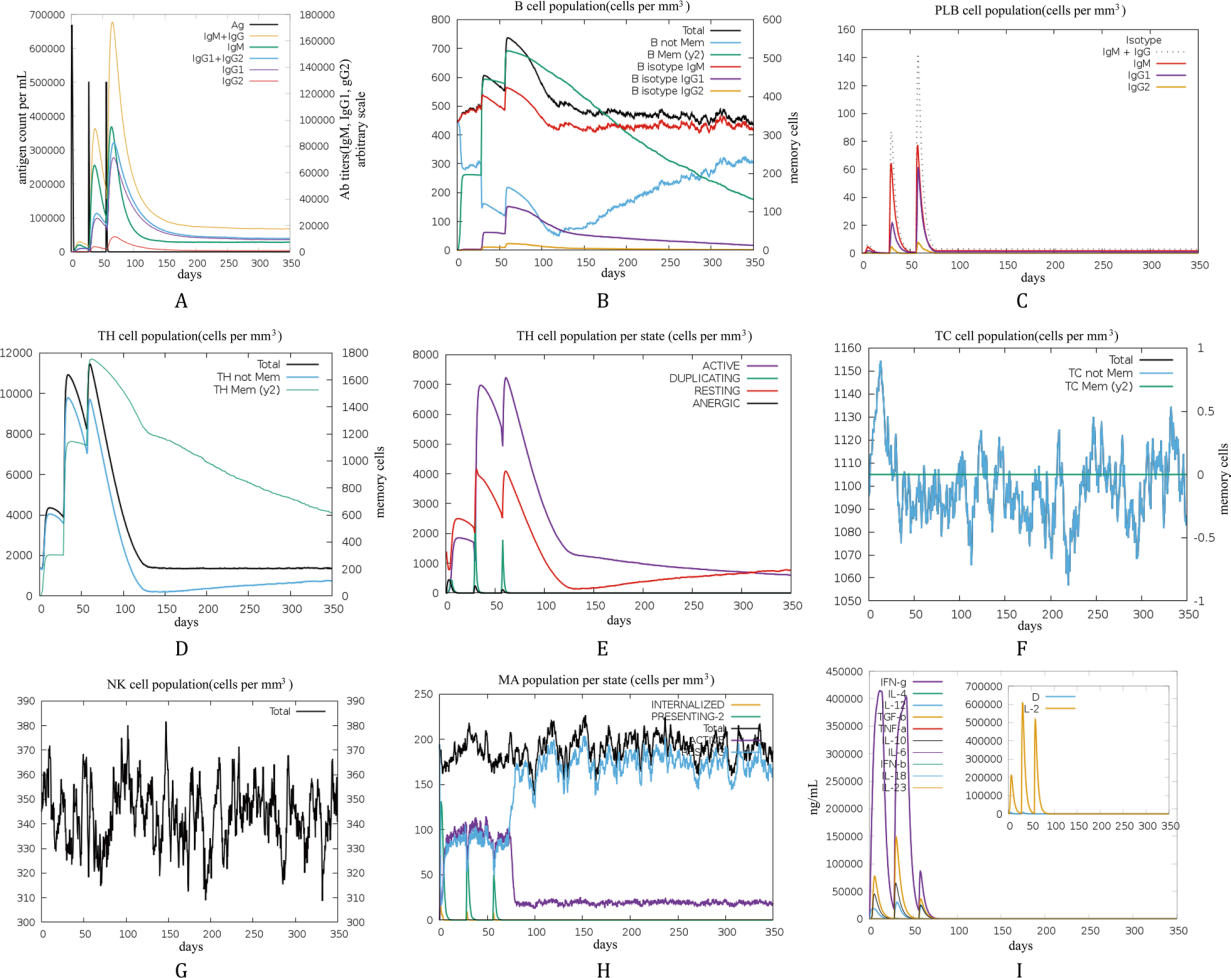
Simulated post-vaccination immune responses. (**A**) Induced antibody titers; (**B**) Induced B cell levels; (**C**) Induced plasma cell counts; (**D**) Induced helper T (Th) cell levels; (**E**) Populations of Th cell subsets across different states; (**F**) Induced cytotoxic T lymphocyte levels; (**G**) NK cell counts; (**H**) Macrophage counts; (**I**) Induced cytokine secretion levels

Immunization simulations were performed using the C-ImmSim server over a 350-day schedule (Fig. 8). Overall, the simulations suggested that the designed vaccine construct could induce sustained humoral and cellular immune responses in the modeled immune system. After the primary immunization, modest increases were observed in antibody levels as well as in B-cell- and T-cell–related compartments, including plasma cells and helper T cells. These responses were further boosted following the second and third immunizations, showing a clear recall pattern (Fig. 8A–F). After the third dose (day 56), the combined IgM + IgG signal reached a peak of approximately 6.8 × 10^5^ arbitrary units (a.u.) in the simulation output (Fig. 8A).

In parallel, the simulation predicted increased cytokine responses, including IFN-γ, IL-2, IL-18, IL-10, and TGF-β. Among these, IFN-γ and IL-2 exhibited the most pronounced elevations, with IFN-γ remaining persistently high and peaking above 4.0 × 10^5^ a.u. (Fig. 8I). Notably, all cytokine and antibody readouts from C-ImmSim are reported in arbitrary simulation units and are therefore interpreted in terms of relative dynamics rather than absolute physiological concentrations.

Regarding cellular responses, the simulated peak counts of activated B cells, helper T cells, and cytotoxic T lymphocytes after the third immunization reached approximately 7.0 × 10^2^, 1.1 × 10^4^, and 1.15 × 10^3^ a.u., respectively (Fig. 8B, D, F). In addition, increased recruitment signals were observed for macrophages, natural killer cells, dendritic cells, and epithelial cells, suggesting coordinated innate and adaptive immune activation in the simulated environment (Fig. 8G–H; Fig. S12). Collectively, these in silico immune simulations support the potential of the vaccine candidate to elicit a robust and durable immune response, while warranting further validation in vitro and in vivo.

### Population coverage

In this study, population coverage analysis of the candidate vaccine was performed based on the MHC allele data corresponding to the epitopes incorporated in its design, using the IEDB Population Coverage database. The results showed that the global population coverage of this multi-epitope candidate vaccine reached 98.65%, and the coverage exceeded 90% in most geographic regions, including North America (99.97%) and Europe (99.92%); only a few regions had slightly lower coverage, which still remained above 90%. Meanwhile, the average hit across regions ranged from 2.16 to 3.70, and the minimum number of epitope combinations required for 90% coverage (pc90) ranged from 1.10 to 2.46, suggesting that both the coverage efficiency and redundancy of the epitope combination were favorable. The regional dimension data of the aforementioned population coverage, average_hit, and pc90 are summarized in Table 6, and the corresponding national-level data are detailed in Supplementary Table S5, which collectively support that the candidate vaccine exhibits promising broad-spectrum protective potential.

**Table 6:** Global and regional population coverage metrics

Geographic region	Class combined		
	Coverage	average_hit	pc90
World	98.65%	3.21	1.93
North America	99.97%	3.65	2.35
Europe	99.92%	3.7	2.46
East Africa	97.57%	2.71	1.52
Central Africa	97.39%	2.63	1.43
South America	97.31%	3.04	1.5
South Asia	96.89%	2.66	1.35
West Africa	96.18%	2.79	1.43
Oceania	94.77%	2.69	1.29
Northeast Asia	93.22%	2.56	1.15
East Asia	92.73%	2.77	1.23
North Africa	92.42%	2.16	1.1

### Construction of multi-epitope mRNA vaccine and in silico cloning

**Fig. 9:**
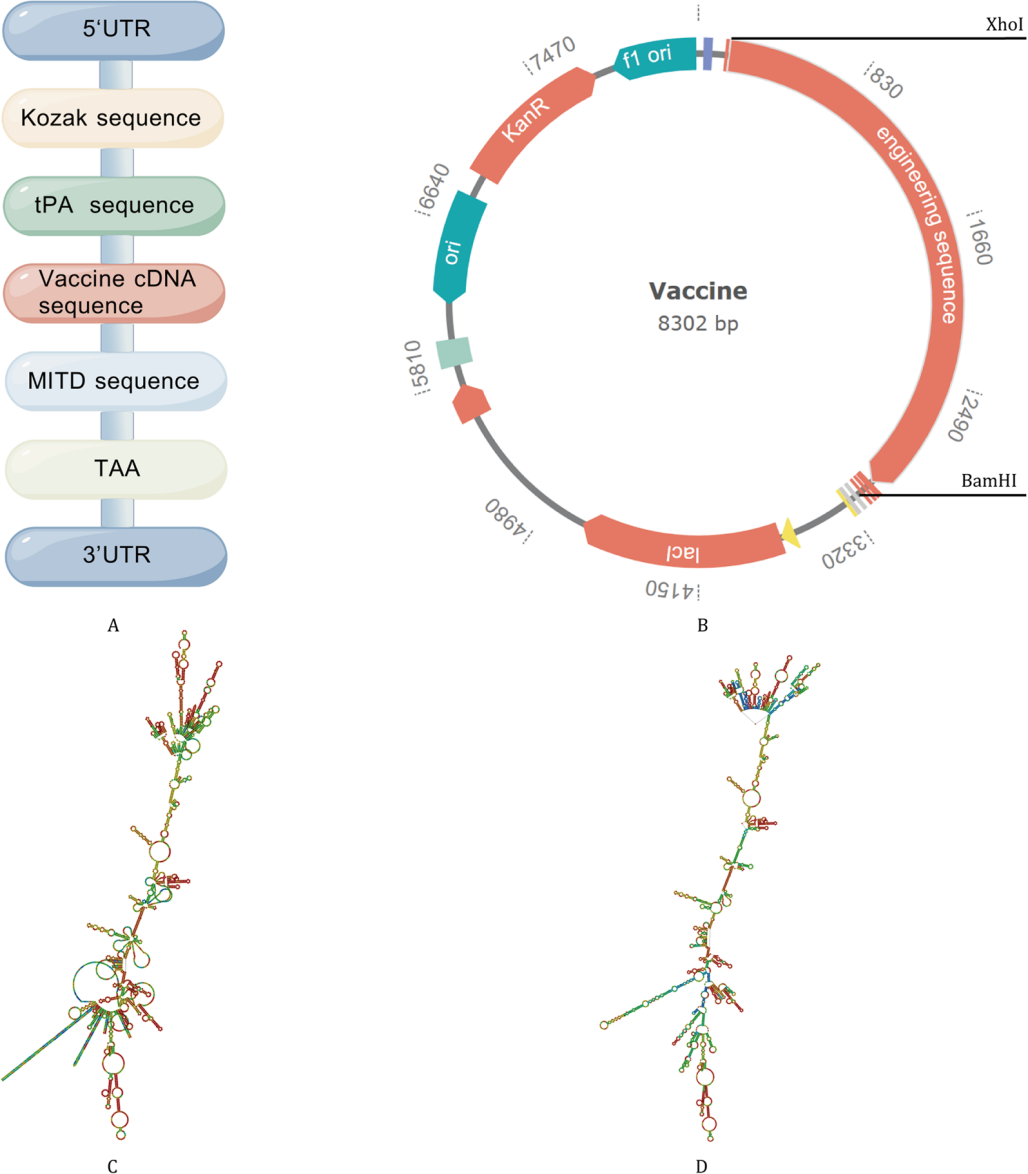
Codon optimization, in silico cloning, and secondary structure prediction for mRNA vaccines. (**A**) Final cDNA sequence construction scheme; (**B**) Plasmid vector construction; (**C**) Minimum free energy secondary structure; (**D**) Central secondary structure

For the final vaccine DNA construct, multiple core sequence modules were integrated, including 5'UTR, 3'UTR, tPA sequence, Kozak sequence, MITD sequence, TAA stop codon, and the cDNA sequence reverse-transcribed from the vaccine’s protein sequence (Fig. 9A). To assess the expression capacity of this DNA sequence in the host strain Escherichia coli K12, this study conducted codon optimization via the JCAT online server, which generated two important metrics: CAI and GC content. The results show that the optimized vaccine sequence exhibited a CAI value of 0.969, indicating efficient heterologous expression in this host system. Additionally, its GC content was measured at 48.26%, falling within the acceptable range of 40–60% for stable gene expression.

Building on these optimization results, the refined sequence was cloned into the pET-28a (+) expression vector via the GenSmart^™^ Design server (Fig. 9B). Furthermore, structural prediction of the mRNA vaccine via the RNAfold server indicated that the minimum free energy of the mRNA’s optimal secondary structure was − 832.60 kcal/mol, whereas the free energy of its centroid secondary structure was − 630.18 kcal/mol (Fig. 9C, D). These data collectively verify that the mRNA vaccine possesses a highly stable secondary structure, supporting its potential for practical translational development.

## Discussion

Diarrheal diseases remain a critical global public health challenge. Among viral pathogens, norovirus, rotavirus, and adenovirus 40/41 are key drivers of morbidity and mortality, particularly in children under 5 years in LMICs [109, 110]. While rotavirus vaccine development has advanced, incomplete cross-genotype protection, lack of licensed norovirus vaccines, and preclinical-stage adenovirus vaccines highlight the urgent need for novel preventive strategies.

The rapid advancement of immunoinformatics technology addresses traditional vaccine development bottlenecks. Integrating reverse vaccinology, pan-genomics, and computational structural biology, it enables precise, high-throughput screening of antigenic targets and epitopes—shortening research cycles while improving vaccine specificity and broad-spectrum efficacy, as validated in landmark studies on herpes simplex virus type 1 and human cytomegalovirus [111, 112]. These methods reduce cumbersome experimental steps in traditional antigen preparation and enhance vaccine efficacy/safety by optimizing epitopes, adjuvants, and linkers—providing an efficient paradigm for addressing unmet needs in viral vaccine development, including multi-pathogen coverage and broad-spectrum protection.

Existing diarrheal virus multi-epitope vaccine studies have laid foundational groundwork: a norovirus-focused study developed a recombinant multi-epitope vaccine, established a broad-spectrum B/T cell epitope screening workflow, and verified immunogenicity in mice [113]; another constructed a bivalent norovirus-rotavirus vaccine via epitope screening and in silico immune potential prediction [114]. However, these studies do not adequately address multiviral co-infections and viral mutations, with two key limitations: restricted target range (excluding adenovirus 40/41, the third major infantile diarrhea virus) and inadequate evaluation systems. To address this, the present study innovates by expanding the pathogen spectrum and conducting comprehensive, systematic assessments of vaccine conservation and safety.

The trivalent multi-epitope mRNA vaccine designed herein mitigates these limitations. Its unique trivalent design offers a promising strategy for norovirus/rotavirus/adenovirus 40/41 co-infections—an unmet clinical challenge for single-pathogen vaccines. Six screened target proteins showed substantial antigenic potential, with no significant toxicity, allergenicity, or transmembrane domains. Epitope screening yielded 16 CTL, 5 HTL, and 17 LBL epitopes, all validated in silico for antigenicity/immunogenicity without safety risks.

For epitope conservation analysis, while self-developed Java-based algorithms offer customization [115], they are hindered by long development cycles, high technical barriers, and poor compatibility with mainstream immunoinformatic tools (e.g., IEDB, MixMHCpred). Based on the IEDB framework, this study established a "basic prediction-secondary validation" system using "IEDB Conservation Analysis Tool + Blastp dual verification," offering three key benefits: low-cost, high-accuracy cross-validated classical methods with broad applicability; comprehensive validation via amino acid identity, entropy, and E-value (confirming conservation and homology); and strong compatibility integrating IEDB tools with MixMHCpred v3.0 to improve prediction accuracy. Limitations include reduced customization flexibility compared to dedicated tools and Blastp sensitivity to pathogen subtype library completeness (potentially biasing new variant assessments). We recommend this "mainstream tool-based" strategy for conserved epitope screening, as it balances efficiency, reliability, and biosafety. Future research could integrate machine learning to optimize tool weight matrices for highly variable pathogens.

In addition, epitope variation arising from viral evolution remains a major barrier to the long-term effectiveness of vaccines—especially for RNA viruses with high mutation rates (e.g., norovirus). To mitigate this risk, we propose four synergistic strategies: (1) screening highly conserved epitopes with homology > 90% and entropy < 0.5 from proteins of different viral subtypes to construct a "conserved core module" accounting for approximately 70%; (2) designing the vaccine as a "conserved core-subtype-specific variable module" (with the variable module accounting for about 30%) to enable rapid replacement of variant epitopes; (3) proposing a "triggered" update mechanism based on quarterly monitoring of global epidemic strains; (4) leveraging the flexibility of mRNA vaccines to enhance iteration efficiency.

The present study has inherent limitations, as it relies solely on immunoinformatics and computational simulations. Built on simplified assumptions, these models cannot fully recapitulate complex, dynamic in vivo biological processes, specifically: (1) Simulations did not account for mRNA construct stability in physiological microenvironments, nuclease degradation risk, or LNP-mediated intracellular delivery efficiency; (2) Target cell translation efficiency—regulated by codon optimization, 5' cap structure, and poly (A) tail length—lacks experimental validation of these key parameters; (3) Inter-host immune response heterogeneity (e.g., TLR expression, antigen-presenting cell function, adaptive immune memory) is not fully captured by computational models; (4) Potential off-target effects and immunotoxicity require direct in vitro/in vivo assessment, as they cannot be predicted here. In summary, the trivalent mRNA vaccine’s predicted immunogenicity, safety, and protective efficacy remain theoretical, requiring systematic validation via sequential in vitro/in vivo experiments and clinical trials.

To address the aforementioned limitations, we have designed a sequential experimental validation plan. Planned in vitro experiments include: (1) TLR activation assay (HEK293-TLR2 stable cell lines + luciferase reporter) to evaluate innate immune signaling; (2) antigen presentation assay (moDC + flow cytometry for MHC-II/CD80/CD86) to verify antigen recognition; (3) cytokine profile assay (PBMC + ELISA) to quantify Th1/Th2 cytokines; (4) LNP vector construction and optimization.

Lipid carrier development is critical for clinical translation. Intestinal-targeted mRNA vaccines face three bottlenecks: nuclease degradation, acid inactivation, and poor mucosal penetration. Insights from a Clostridioides difficile mRNA-LNP vaccine [116]—including four-component LNPs for efficient encapsulation and endosomal escape, intramuscular injection to trigger "systemic-mucosal crosstalk" (activating intestinal mucosal immunity indirectly), and multivalent antigen design—guide our targeted delivery system optimization: (1) optimizing four-component LNP lipid ratios for multi-epitope mRNA, preparing 100–150 nm complexes via microfluidic mixing; (2) adopting "intramuscular injection + multi-epitope antigen" to bypass oral barriers and activate intestinal mucosal immunity; (3) evaluating feasibility via physicochemical characterization (particle size, encapsulation efficiency), in vitro transfection, in vivo immunity, and gut microbiota safety. Subsequent optimizations (e.g., mucosal adjuvants, targeting ligands) will address delivery challenges [117].

The *E. coli* K12-pET-28a (+) prokaryotic system was used for preliminary validation of (1) HLA binding activity (a prerequisite for immune function) and (2) in vitro epitope immunogenicity. Selected for its simplicity, high expression efficiency, and cost-effectiveness, this prokaryotic system enables rapid preliminary screening of recombinant epitope proteins. However, it is constrained by prokaryote-eukaryote differences (e.g., lack of eukaryotic post-translational modifications, distinct codon preferences, and improper protein folding)—thus limiting its utility to basic epitope validation (not for mRNA vaccine production or functional assessment).

For mRNA vaccine production, the rabbit reticulocyte cell-free system (enriched with eukaryotic translation machinery, nuclease-free) will be used. Systematic codon optimization via OptimumGene^™^ will balance parameters: GC content (50%-60%), replacement of rare codons (e.g., AGG, CGA) with high-frequency synonyms, elimination of inhibitory secondary structures (ΔG > − 30 kcal/mol via RNAfold), optimized Kozak sequence conservation, and avoidance of repeats/IRES-like structures. This will generate high-expression mRNA sequences supporting subsequent production and in vivo evaluation.

Finally, future research will prioritize three key directions to advance clinical translation: (1) validating vaccine immunogenicity and mRNA stability in cell models mimicking the intestinal microenvironment; (2) verifying in vivo immune efficacy and intestinal-targeted protection in age-matched animal models (e.g., suckling mice, which recapitulate infant immune characteristics); (3) optimizing delivery systems (e.g., intestinal-targeted LNPs) and integrating multi-omics to dissect immune mechanisms, thereby supporting large-scale production and deployment in resource-limited regions.

## Conclusions

This study computationally designed a trivalent multi-epitope mRNA vaccine targeting norovirus, rotavirus, and adenovirus 40/41. Computational simulation analyses have indicated that the vaccine may exhibit favorable structural stability, potential immune activation capability, and promising coverage across diverse global populations, thereby providing preliminary insights for the development of vaccines against multiple viral co-infections associated with diarrheal diseases. It should be noted that this study still has limitations: the current results are solely based on computational simulation analyses, and the safety and efficacy of the vaccine await verification through animal model experiments and subsequent clinical trials; meanwhile, the optimization of the vaccine delivery system and the improvement of the cell-free production process also require further exploration.

## Supplementary Information

Additional file 1.

Additional file 2.

## Data Availability

All datasets generated or analyzed in the current study are available at https://zenodo.org/records/17927091. The record is publicly accessible, while access to the files is restricted to authorized users. For obtaining the relevant files, please contact via email: 8301200321@csu.edu.cn.

## Abbreviations

LMICs: Low- and middle-income countries
CTL: Cytotoxic T lymphocyte
HTL: Helper T lymphocyte
LBL: Linear B lymphocyte
IEDB: Immune Epitope Database
HLA: Human leukocyte antigen
TLRs: Toll-like receptors
DC: Dendritic cell
MD: Molecular dynamics
RMSD: Root mean square deviation
RMSF: Root mean square fluctuation
Rg: Radius of gyration
MM-PBSA: Molecular mechanics/Poisson-Boltzmann surface area
CAI: Codon Adaptation Index
*SD*: Standard deviation
