# Supplementary material for: Trivalent multi-epitope mRNA vaccine against norovirus, rotavirus, and adenovirus 40/41: epitope screening, molecular docking, and molecular dynamics simulation with in silico validation guided by immunoinformatics
Source: Infect Dis Poverty. 2026 Mar 27;15:36. doi: 10.1186/s40249-026-01431-y (PMC13023124; doi:10.1186/s40249-026-01431-y)
Supplement: Supplementary file 1 — Additional file 1. [file 40249_2026_1431_MOESM1_ESM.docx]

Supplementary Material

1. **Supplementary Diagram**

**1.1 Supplementary Figure 1**

**
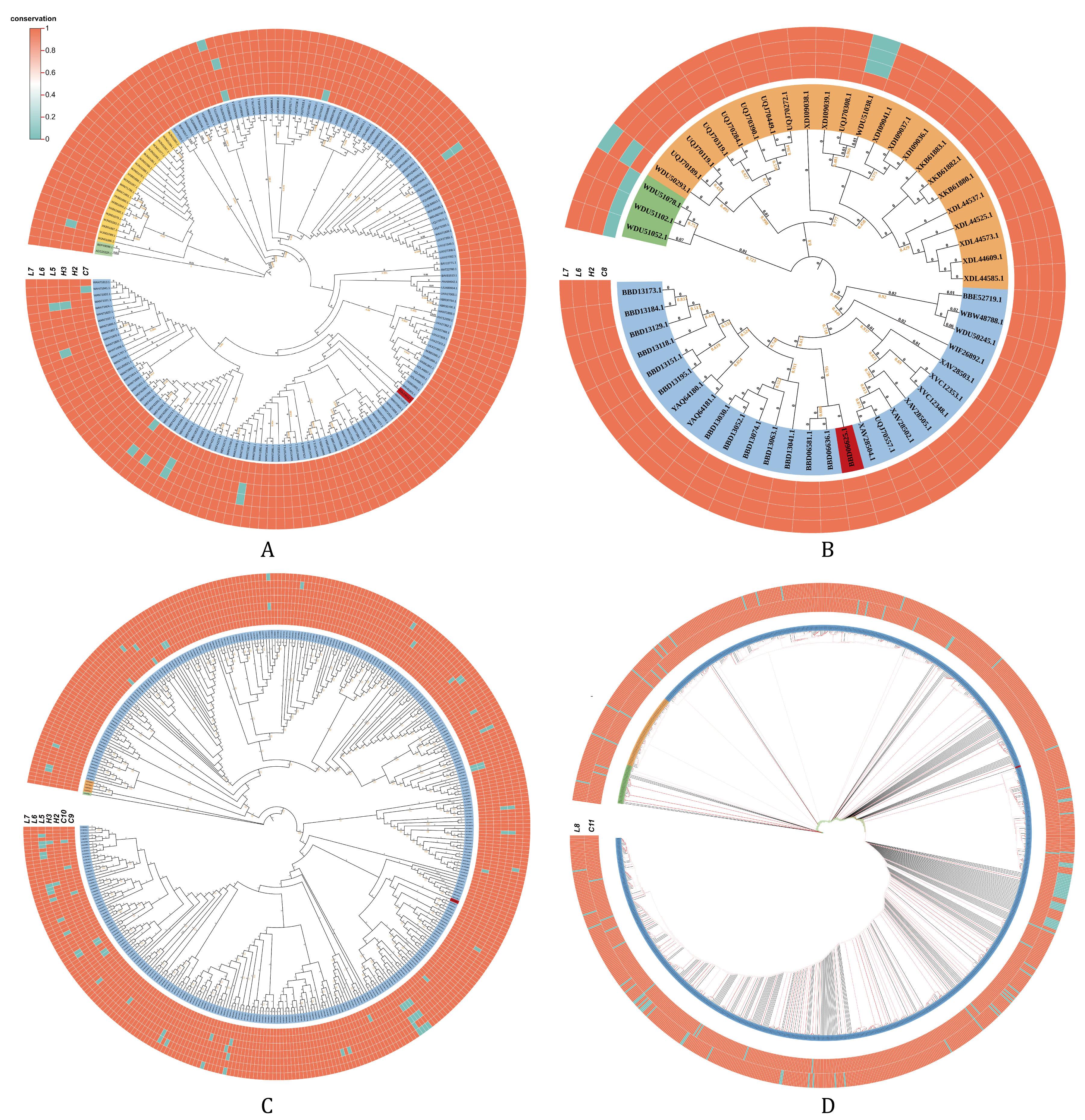
**

**Supplementary Figure 1.** Phylogenetic tree and epitope conservation analysis of VP4 and VP6 proteins from different rotavirus subtypes (Inner circle: Phylogenetic tree, with red sequences representing the target sequences selected for epitope screening; Outer circle: Distribution of epitope conservation). (**A**) P [4] subtype; (**B**) P [6] subtype; (**C**) P [8] subtype; (**D**) Group A of VP6 protein.

**1.2 Supplementary Figure 2**

**
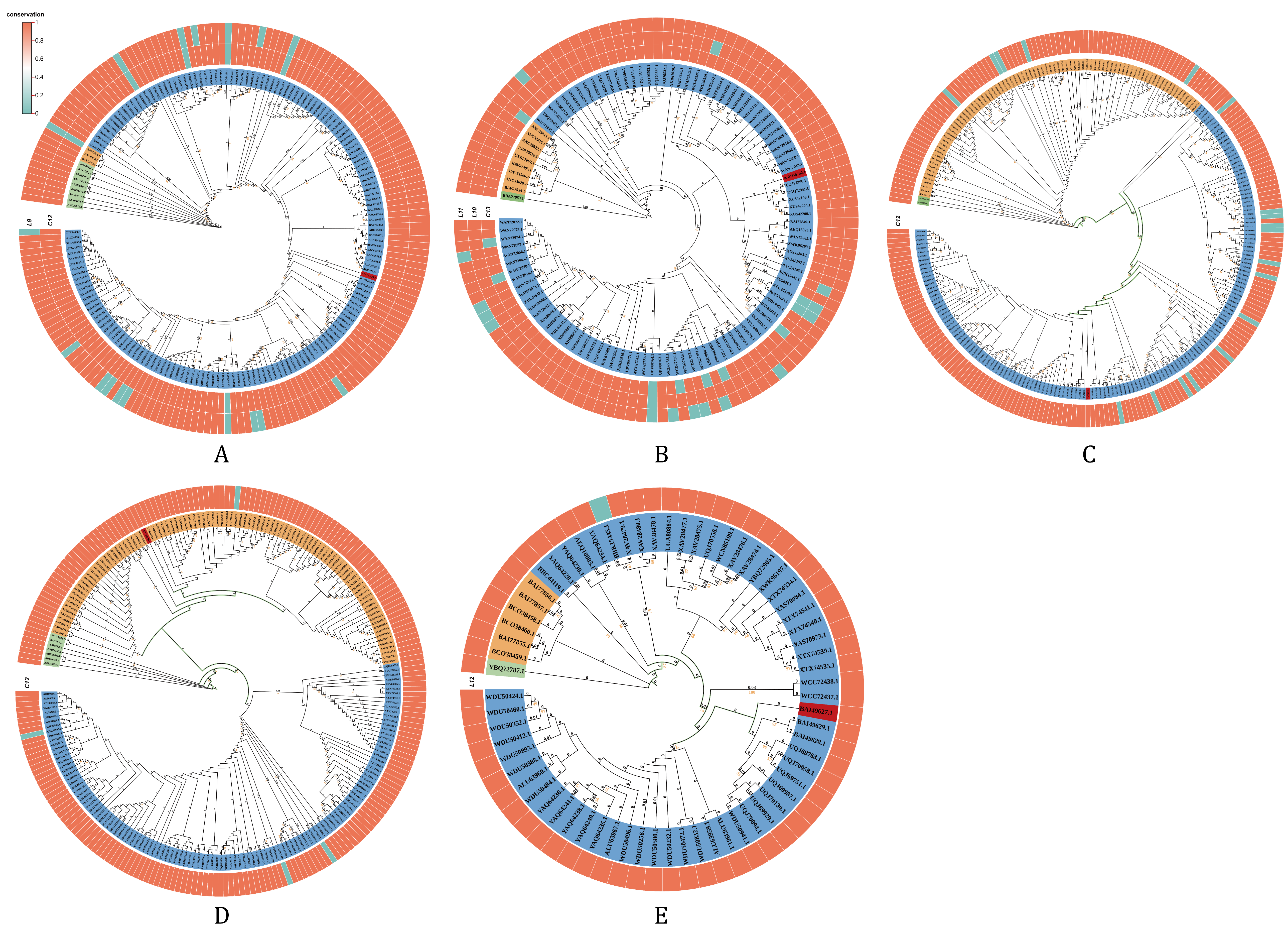
**

**Supplementary Figure 2.** Phylogenetic tree and epitope conservation analysis of VP7 proteins from different rotavirus subtypes (Inner circle: Phylogenetic tree, with red sequences representing the target sequences selected for epitope screening; Outer circle: Distribution of epitope conservation). (**A**) G1 subtype; (**B**) G2 subtype; (**C**) G3 subtype; (**D**) G9 subtype; (**E**) G12 subtype.

**1.3 Supplementary Figure 3**

**
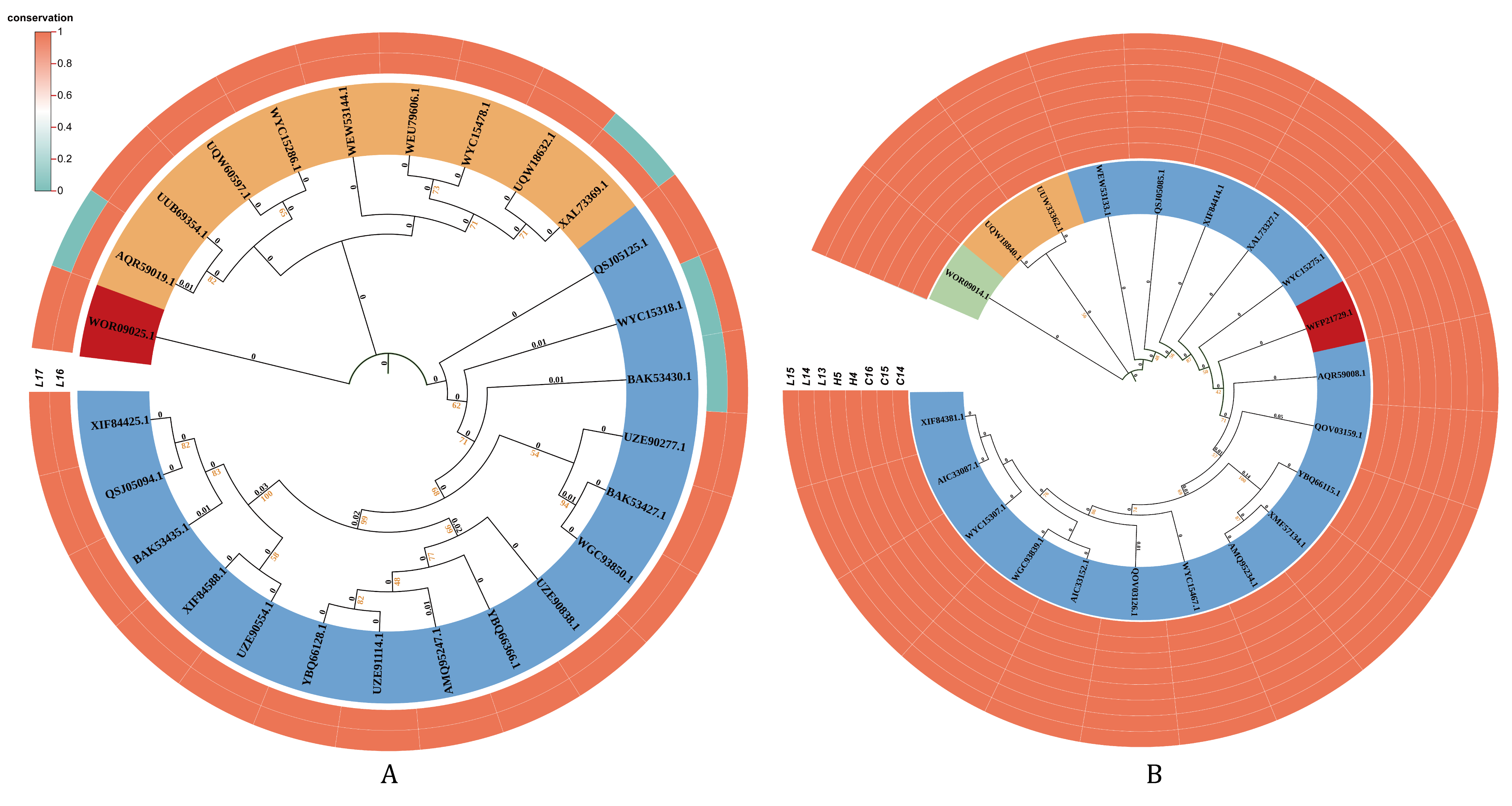
**

**Supplementary Figure 3.** Phylogenetic tree and epitope conservation analysis of hexon and short fiber proteins from adenovirus 40/41 subtypes (Inner circle: Phylogenetic tree, with red sequences representing the target sequences selected for epitope screening; Outer circle: Distribution of epitope conservation). (**A**) Hexon protein; (**B**) Short fiber protein.

**1.4 Supplementary Figure 4**

**
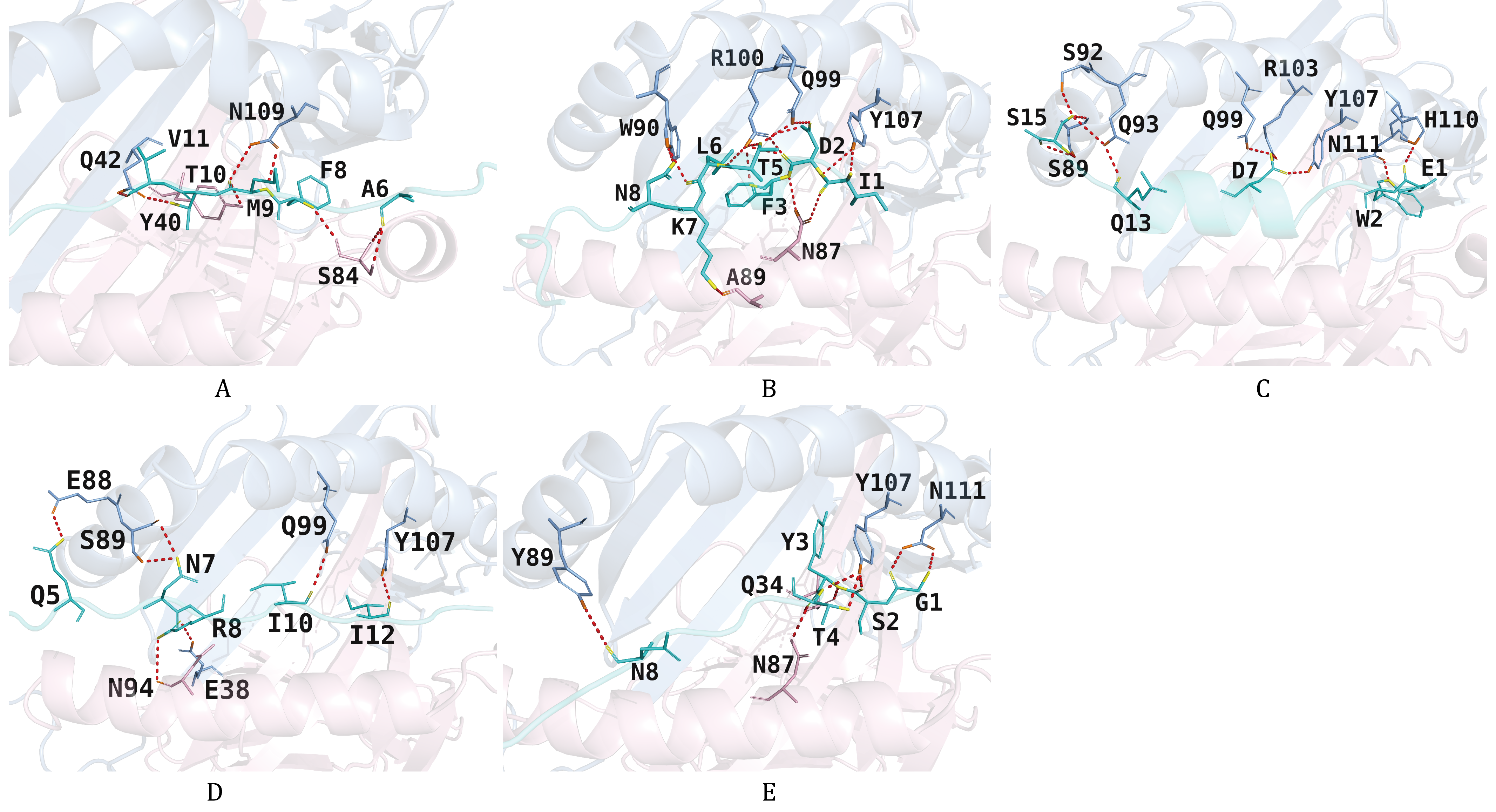
**

**Supplementary Figure 4.** The Schematic diagram of docking between each of the 5 HTL epitopes and HLA molecules.

**1.5 Supplementary Figure 5**

**
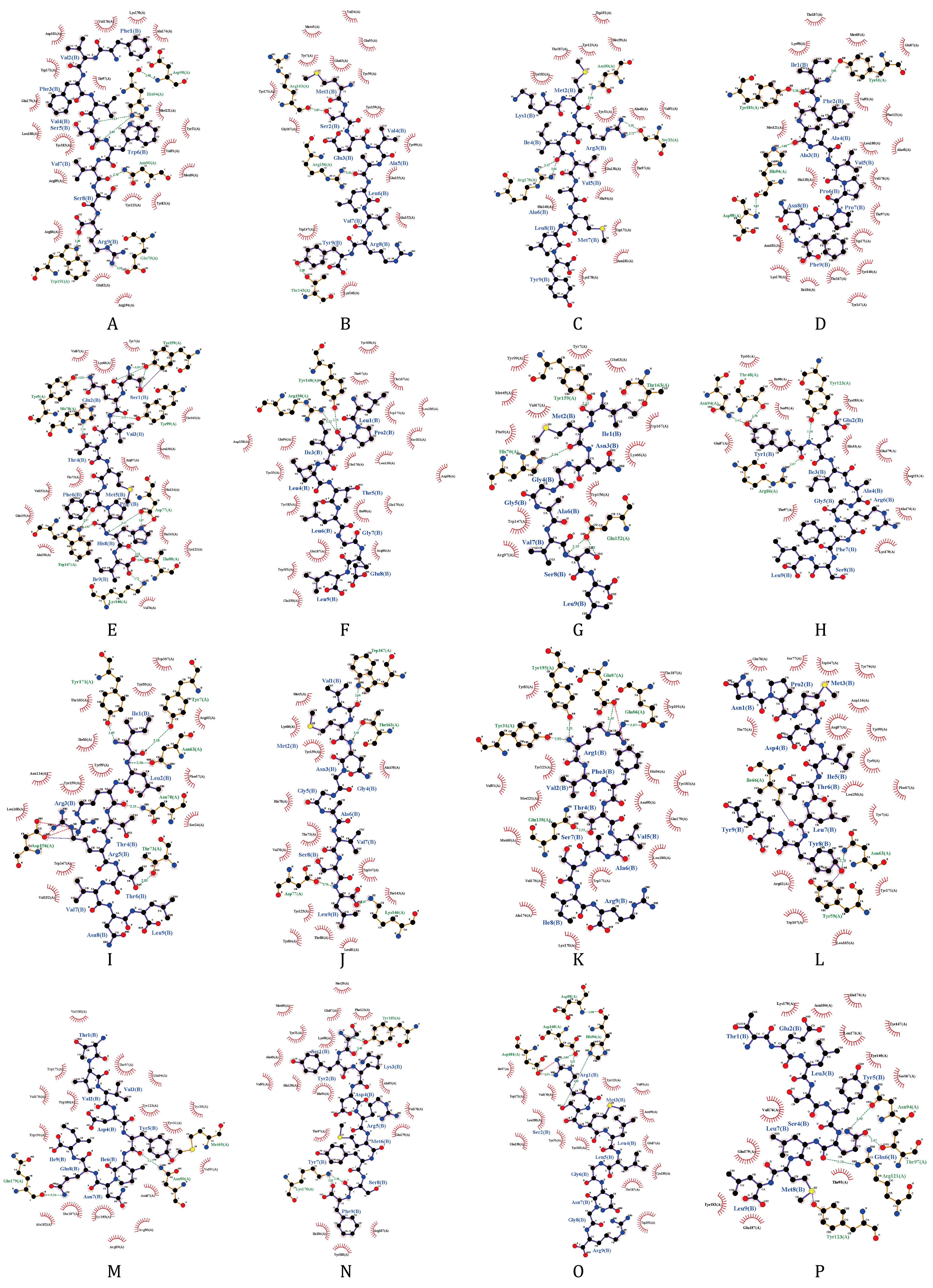
**

**Supplementary Figure 5.** Analysis of Interaction Characteristics Between CTL Epitopes and HLA Molecules Using LigPLOT^+^ TOOLs.

**1.6 Supplementary Figure 6**

**
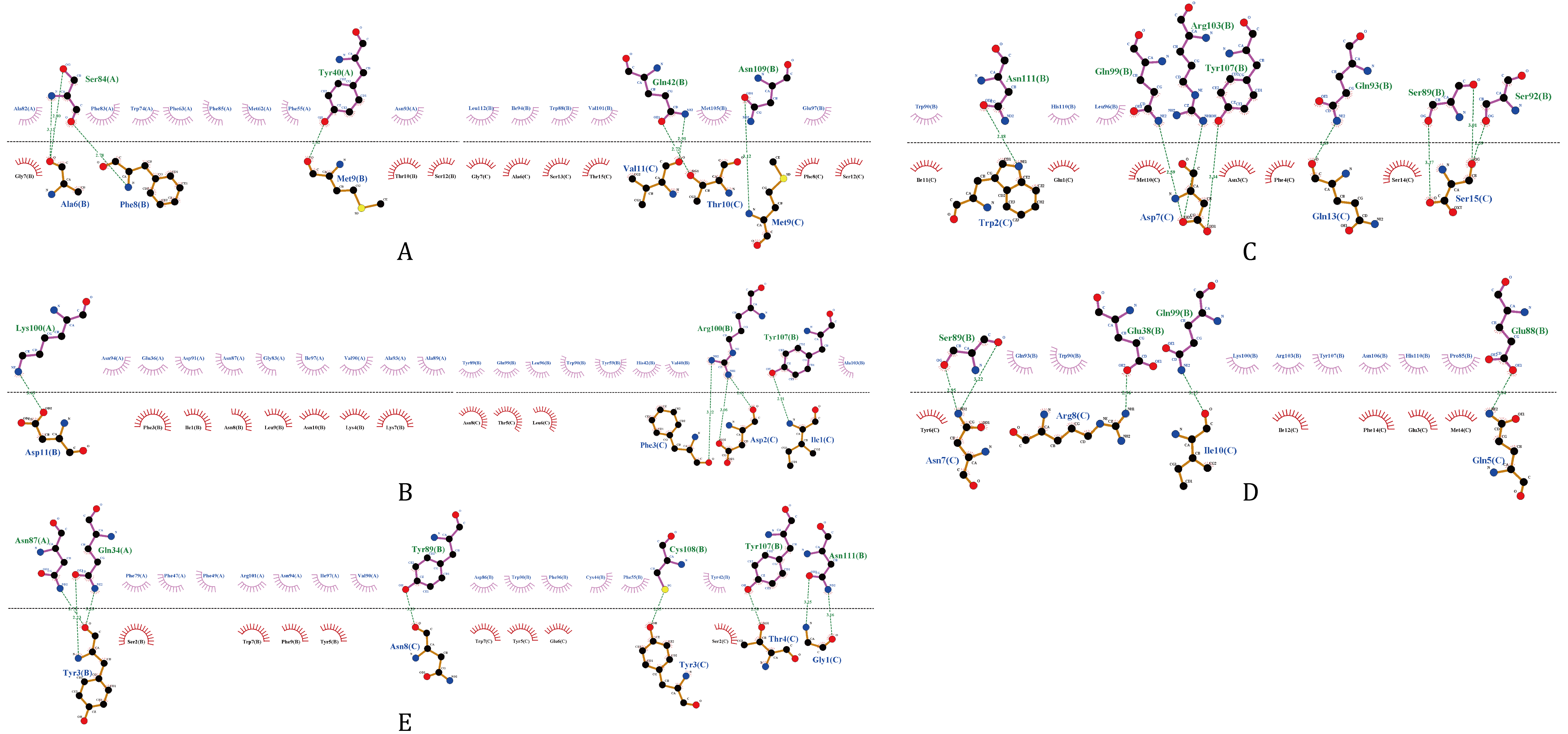
**

**Supplementary Figure 6.** Analysis of Interaction Characteristics Between HTL Epitopes and HLA Molecules Using LigPLOT+ TOOLs.

**1.7 Supplementary Figure 7**

**
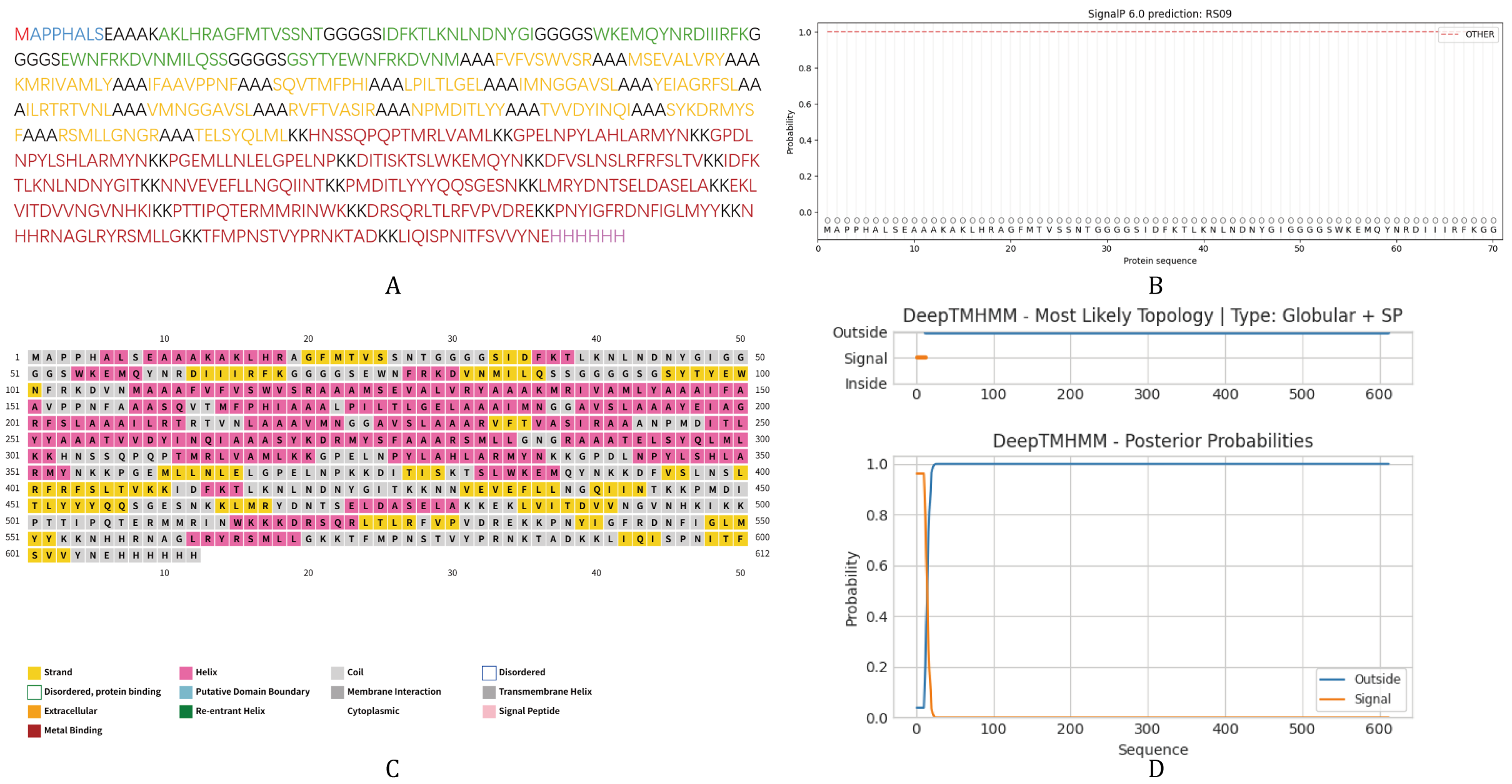
**

**Supplementary Figure 7.** Vaccine Constructed with RS09 Adjuvant: Analysis of (**A**)Sequence; (**B**)Signal Peptide; (**C**)Secondary Structure; (**D**)Transmembrane Helices.

**1.8 Supplementary Figure 8**

**
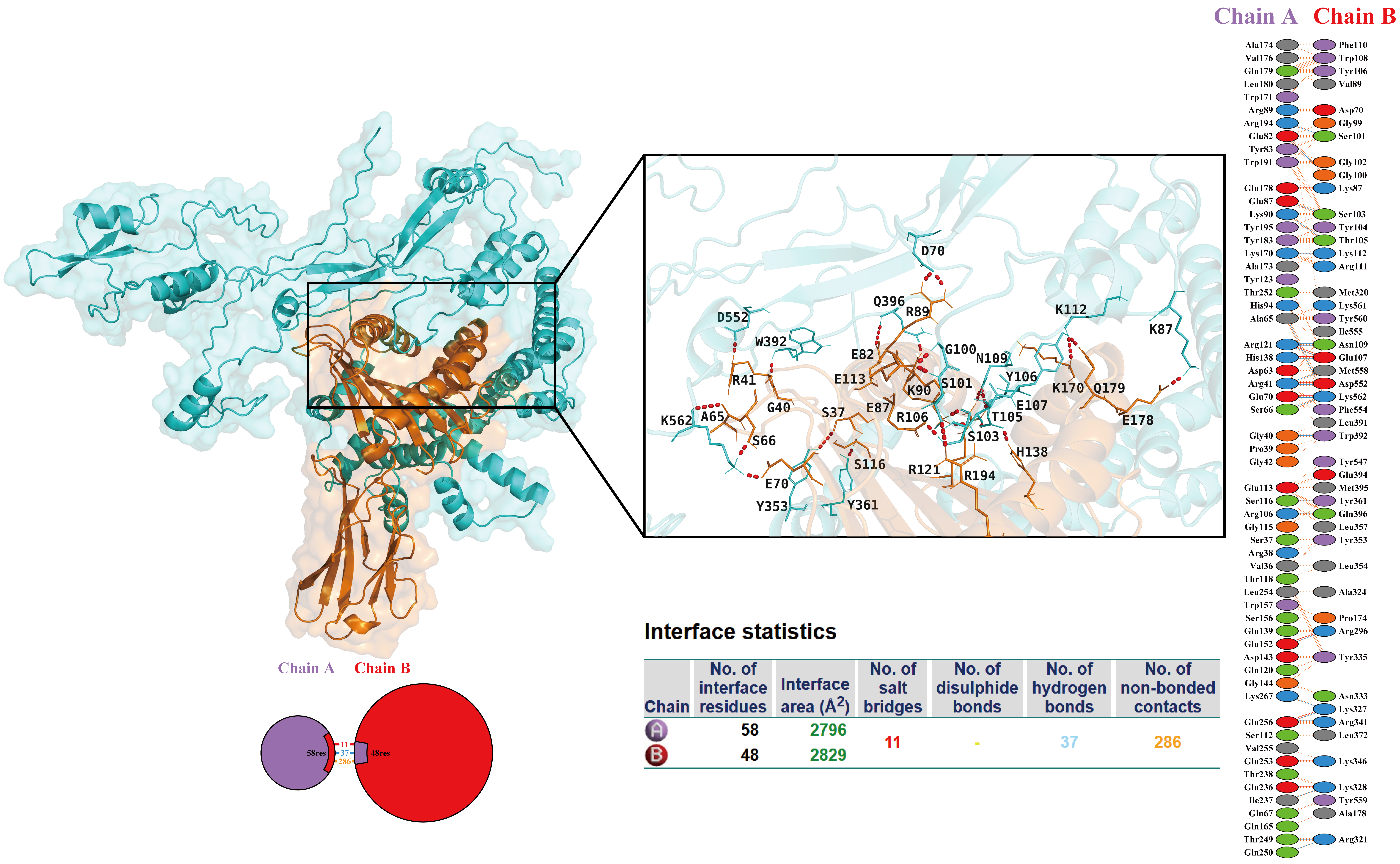
**

**Supplementary Figure 8.** Results of Vaccine-HLA-A*02:01 Molecule Docking.

**1.9 Supplementary Figure 9**

**
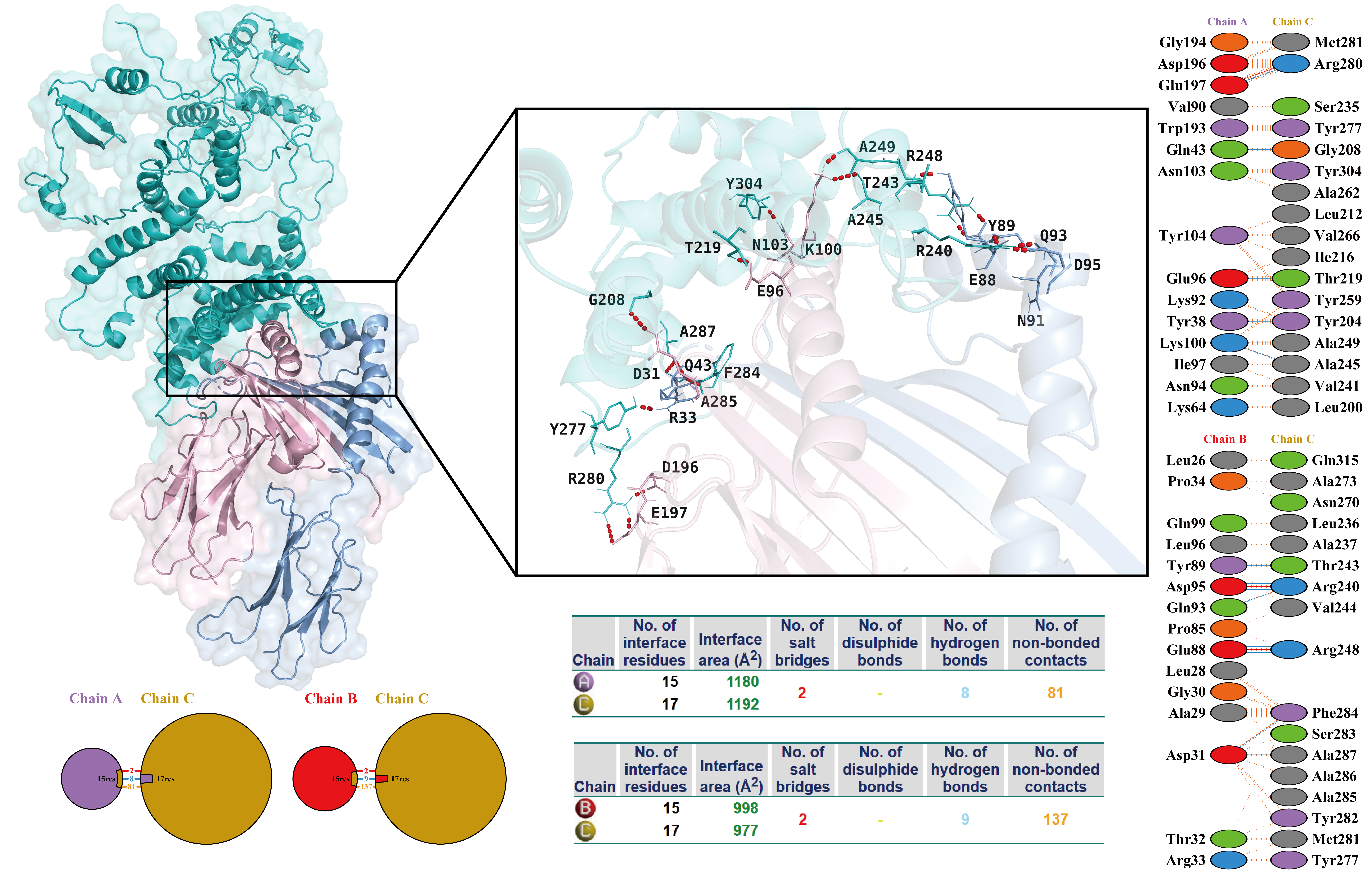
**

**Supplementary Figure 9.** Results of Vaccine-HLA-DRB1*01:01 Molecule Docking.

**1.10 Supplementary Figure 10**

**
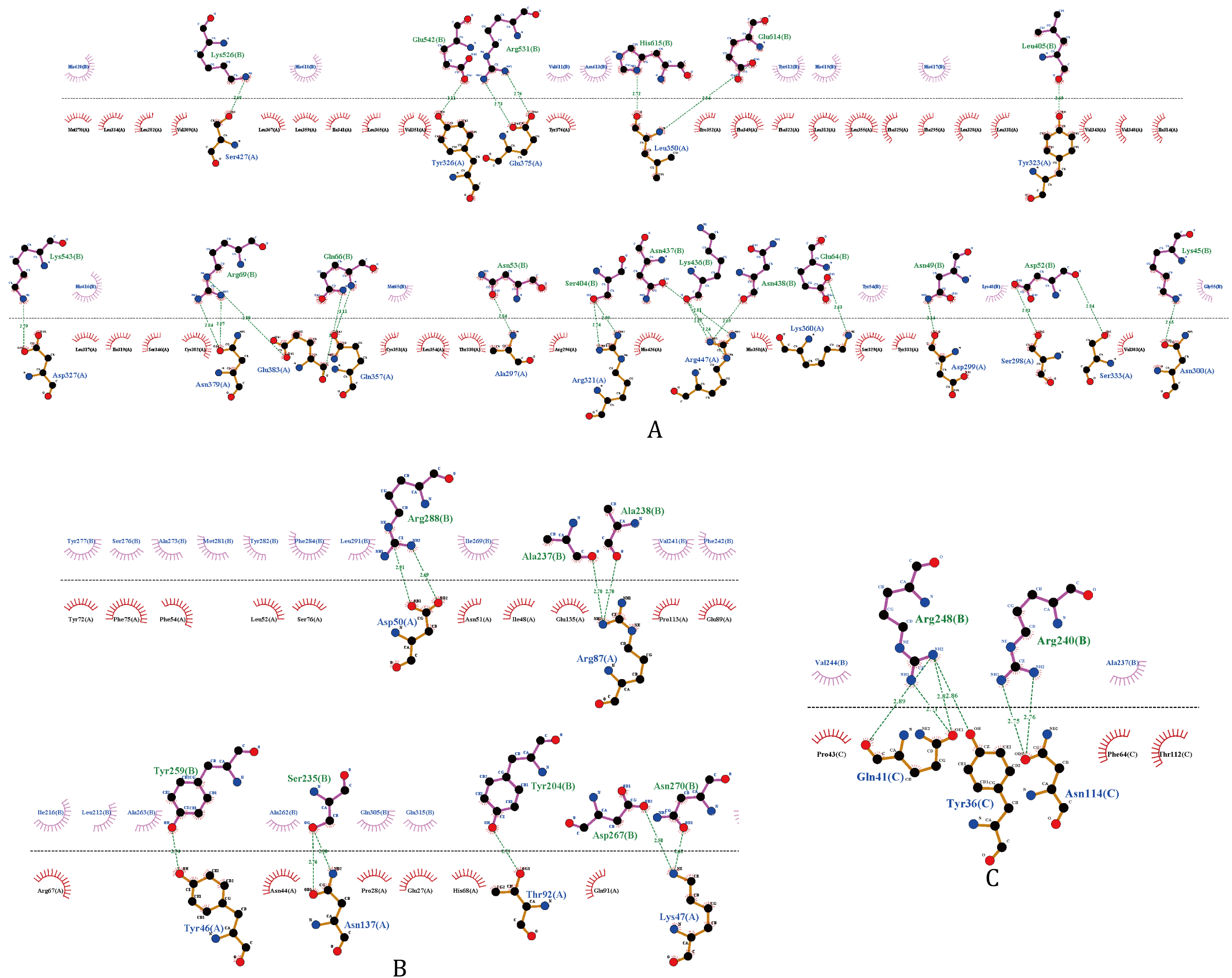
**

**Supplementary Figure 10.** Analysis of Interaction Characteristics Between Vaccine and HLA-A*02:01 Molecules Using Ligplot+ Tool.

**1.11 Supplementary Figure 11**

**
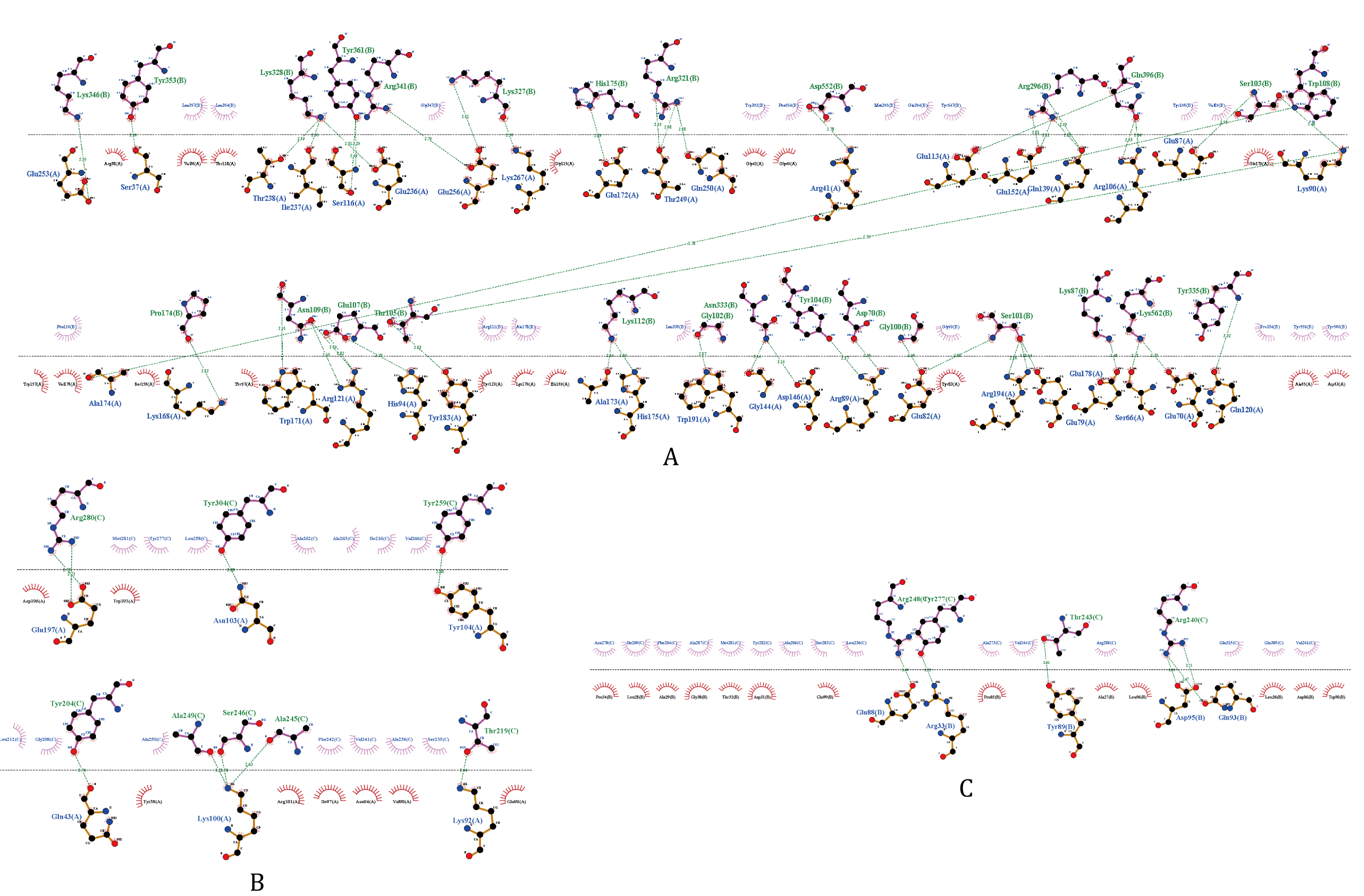
**

**Supplementary Figure 11.** Analysis of Interaction Characteristics Between Vaccine and HLA-DRB1*01:01 Molecules Using Ligplot+ Tool.

**1.12 Supplementary Figure 12**

**
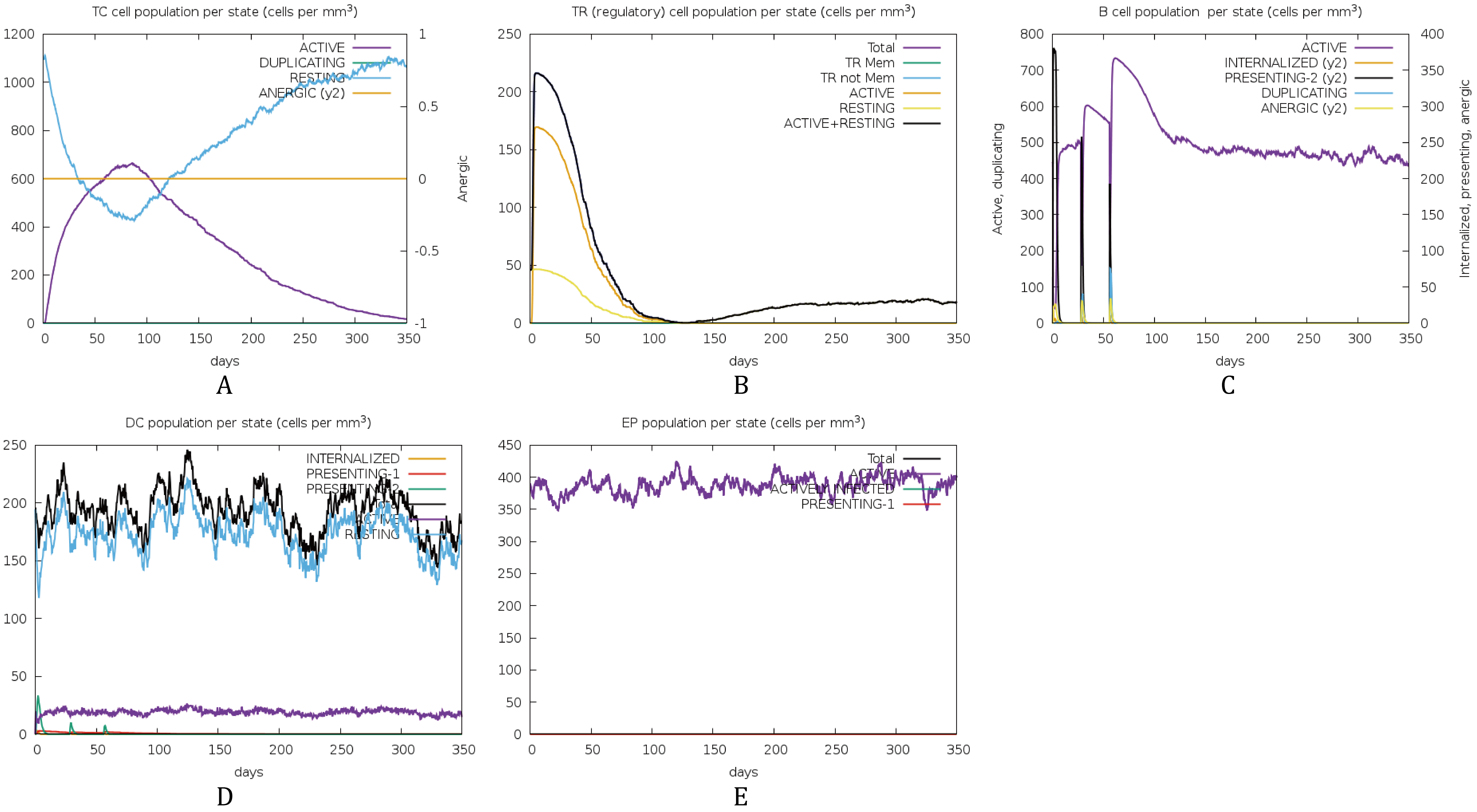
**

**Supplementary Figure 12.** Other Immune Simulation Result Figures: Display of TH Cells, TR Cells, B Cells, DC Cells and EP Levels at Different Periods.

1. **Supplementary Data**

**2.1 Target protein sequences of NV, RV and AV：**

**VP1:**

>WYW48828.1 |VP1 [Norovirus GI.3]

MMMASKDAPTNMDGTSGAGQLVPEANTAEPISMEPVAGAATAAATAGQVNMIDPWIMNNYVQAPQGEFTISPNNTPGDILFDLQLGPHLNPFLSHLAQMYNGWVGNMKVKVLLAGNAFTAGKIIISCIPPGFAAQNISIAQATMFPHVIADVRVLEPIEVPLEDVRNVLFHNNDNTPTMRLVCMLYTPLRASGSSSGTDPFVIAGRVLTCPSPDFSFLFLVPPNVEQKTKPFSVPNLPLNTLSNSRVPSLIRSMMISRDHGQMVQFQNGRVTLDGQLQGTTPTSASQLCKIRGSVFHANGGNGFNLTELDGSPYHAFESPAPIGFPDLGECDWHMEASPTTQFDTGDVIKQINVKQESAFAPHLGTVQADGLSDVSANTNMIAKLGWVSPVSDGHRRDVDPWVIPRYGSTLTEAAQLAPPIYPPGFGEAIVFFMSDFPIAHGTNGLSVPCTIPQEFVTHFVNEQAPTRGEAALLHYLDPDTHRNLGEFKLYPDGFMTCVPNSSGTGPQTLPINGVFVFVSWVSRFYQLKPVGTAGPARRLGIRRS

>AFH88383.1 |VP1 [Norovirus Hu/GI.6/Kingston/ACT160D/2010/AU]

MMMASKDAPTSPDGASGAGQLVPEANTAEQISMDPVAGASTAVATAGQVNMIDPWIFNNFVQAPQGEFTISPNNTPGDILFDLQLGPHLNPFLAHLSQMY NAFTAGKIIICCVPPGFDARILTIAQATLFPHLIADVRTLEPVELPLEDVRNVLYHNSSQPQPTMRLVAMLYTPLRTGGGSGGTDAFVVAGRVLTCPAPDFSFLFLVPPSVEQKTRVFSVPNIPLKDLSNSRVPVPVQGMFMSPDVNQSVQFQNGRCQIDGQLQGTTPVSLSQLCKIRGKTSSNARVLNLSEVDGTPFIPLESPAPVGFPDLGGCDWHVNFTFQAQNQDPSQSVTFATNDASFVPYLGSISPHNGGDFHAGDIIGSLGWISAPSDNSQLNVWTIPKYGSSLPDVTHLAPAVFPPGFGEVILYFYSTFPGSGQPSQLQVPCLLPQEFITHFCNEQAPIAGEAALLHYVDPDTGRNLGEFKLYPDGFMTCVPNSVSSGPQTLPINGVFVFVSWVSRFYQLKPVGTASAARRLGLRRI

>AUD39963.1 |VP1 [Norovirus GII.P16/GII.2]

MKMASNDAAPSTDGAAGLVPESNNEVMALEPVAGAALAAPVTGQTNIIDPWIRANFVQAPNGEFTVSPRNAPGEVLLNLELGPELNPYLAHLARMYNGYAGGMEVQVMLAGNAFTAGKLVFAAVPPHFPVENLSPQQITMFPHVIIDVRTLEPVLLPLPDVRNNFFHYNQKDDPKMRIVAMLYTPLRSNGSGDDVFTVSCRVLTRPSPDFDFTYLVPPTVESKTKPFTLPILTLGELSNSRFPVSIDQMYTSPNEVISVQCQNGRCTLDGELQGTTQLQVSGICAFKGEVTAHLHDNDHLYNVTITNLNGSPFDPSEDIPAPLGVPDFQGRVFGIISQRDKHNSPGHNEPANRGHDAVVPTYTAQYTPKLGQIQIGTWQTDDLTVNQPVKFTPVGLNDTEHFNQWVVPRYAGALNLNTNLAPSVAPVFPGERLLFFRSYIPLKGGYGNPAIDCLLPQEWVQHFYQEAAPSMSEVALVRYINPDTGRALFEAKLHRAGFMTVSSNTSAPVVVPANGYFRFDSWVNQFYSLAPMGTGNGRRRVQ

>ANP93502.1 |VP1 [Norovirus GII.4]

MKMASSDANPSDGSAANLVPEVNNEVMALEPVVGAAIAAPVAGQQNVIDPWIRNNFVQAPGGEFTVSPRNAPGEILWSAPLGPDLNPYLSHLARMYNGYAGGFEVQVILAGNAFTAGKIIFAAVPPNFPTEGLSPSQVTMFPHIIVDVRQLEPVLIPLPDVRNNFYHYNQSNDPTIKLIAMLYTPLRANNAGDDVFTVSCRVLTRPSPDFDFIFLVPPTVESRTKPFSVPILTVEEMTNSRFPIPLEKLFTGPSSAFVVQPQNGRCTTDGVLLGTTQLSPVNICTFRGDVTHITGSRNYTMNLASQNWNNYDPTEEIPAPLGTPDFVGKIQGVLTQTTRADGSTRGHKATVYTGSADFAPKLGRVQFETDTDHDFEANQNTKFTPVGVIQDGGTTHRNEPQQWVLPSYSGRNTHNVHLAPAVAPTFPGEQLLFFRSTMPGCSGYPNMDLDCLLPQEWVQYFYQEAAPAQSDVALLRFVNPDTGRVLFECKLHKSGYVTVAHTGQHDLVIPPNGYFRFDSWVNQFYTLAPMGNGTGRRRAV

>ANY58562.1 |major capsid protein [Norovirus Hu/GII.P7_GII.6/Maryland/2014/USA]

MKMASNDAAPSNDGAANLVPEATNEVMALEPVVGASIAAPVVGQQNIIDPWIRENFVQAPQGEFTVSPRNSPGEMLLNLELGPELNPYLSHLSRMYNGYAGGMQVQVVLAGNAFTAGKIIFAAVPPHFPVENISAAQITMCPHVIVDVRQLEPVLLPLPDIRNRFFHYNQENTPRMRLVAMLYTPLRANSGEDVFTVSCRVLTRPAPDFEFTFLVPPTVESKTKPFTLPILTLGELSNSRFPAPIDMLYTDPNEAIVVQPQNGRCTLDGTLQGTTQLVPTQICSFRGTLISQTSRSADSTDSAPRARNHPLHVQLKNLDGTPYDPTDEVPAVLGAIDFKGTVFGVASQRNTTGNSIGATRAHEVHIDTTNPRYTPKLGSVLMHSESTDFDDGQPTRFTPIGMGADDWHQWELPEYSGHLTLNMNLAPAVAPAFPGERILFFRSVVPSAGGYGSGHIDCLIPQEWVQHFYQEAAPSQSAVALIRYVNPDTGRNIFEAKLHREGFITVANSGNNPIVVPPNGYFRFEAWVNQFYTLTPMGTGQGRRRVQ

**VP4:**

>XDL44633.1 |VP4 [Rotavirus A P[4]]

MASLIYRQLLTNSYSVDLHDEIEQIGSEKTQNVTVNPGPFAQTRYAPVNWGHGEINDSTTVEPVLDGPYQPTTFKPPNDYWLLISSNTDGVVYESTNNSDFWTAVIAVEPHVSQTNRQYVLFGENKQFNIENSSDKWKFFEMFKGSNQSDFSNRRTLTSNNRLVGMLKYGGRVWTFHGETPRATTDSSNTADLNNISIIIHSEFYIIPRSQESKCNEYINNGLPPIQNTRNVVPLSLSSRSIQYRRAQVNEDITISKTSLWKEMQYNRDIIIRFKFGNSVIKLGGLGYKWSEISYKAANYQYSYSRDGEQVTAHTTCSVNGVNNFSYNGGSLPTDFSISRYEVIKENSYVYIDYWDDSKAFRNMVYVRSLAANLNSVKCVGGSYDFRLPVGEWPIMNGGAVSLHFAGVTLSTQFTDFVSLNSLRFRFSLTVDEPSFSIIRTRTMNLYGLPAANPNNGNEYYEVSGRFSLISLVPTNDDYQTPIMNSVTVRQDLERQLNDLREEFNSLSQEIAMSQLIDLALLPLDMFSMFSGIKSTIDLTKSMATSVMKKFRKSKLATSISEMTNSLSDAASSASRSASIRSNLSTISNWSDASKSVLNVTDSVNDISTQTSTISKKLRLKEMITQTEGISFDDISAAVLKTKIDMSTQIGKNTLPDIVTEASEKFIPKRSYRVLKDDEVMEVNTEGKFFAYKVDTLNEIPFDINKFAELVTDSPVISAIIDFKTLKNLNDNYGITRIEALNLIKSNPNVLRNFINQNNPIIRNRIEQLILQCKL

>BBD06625.1 |viral protein 4 [Rotavirus A P[6]]

MASLIYRQLLTNSYTVELSDEINTIGSEKSQNVTINPGPFAQTNYAPVTWSHGEVNDSTTIEPVLDGPYQPTNFKPPNDYWILLNPTNQQVVLEGTNKTDVWVALLLVEPNVTNQSRQYTLFGETKQITVENNTNKWKFFEMFRNNVSAEFQHKRTLTSDTKLAGFMKFYNSVWTFHGETPHATTDYSSTSNLSEVETVIHVEFYIIPRSQESKCSEYINTGLPPMQNTRNIVPVALSSRSVTYQRAQVNEDIIISKTSLWKEMQYNRDIIIRFKFNNSIVKLGGLGYKWSEISFKAANYQYSYLRDGEQVTAHTTCSVNGVNNFSYNGGSLPTDFSVSRYEVIKENSYVYVDYWDDSQAFRNMVYVRSLAANLNSVKCSGGNYNFQIPVGAWPVMSGGAVSLHFAGVTLSTQFTDFVSLNSLRFRFSLTVEEPPFSILRTRVSGLYGLPAFNPNNGHEYYEIAGRFSLISLVPSNDDYQTPIMNSVTVRQDLERQLGDLREEFNSLSQEIAMTQLIDLALLPLDMFSMFSGIKSTIDVAKSMVTKVMKKFKKSGLATSISELTGSLSNAASSVSRSSSIRSNISSISVWTDVSEQIAGSSDSVRNISTQTSAISKRLRLREITTQTEGMNFDDISAAVLKTKIDRSTHISPDTLPDIITESSEKFIPKRAYRVLKDDEVMEADVDGKFFAYKVGTFEEVPFDVDKFVDLVTDSPVISAIIDFKTLKNLNDNYGITRSQALDLIRSDPRVLRDFINQNNPIIKNRIEQLILQCRL

>XQU58909.1 |VP4 [Rotavirus A P[8]]

MASLIYRQLLTNSYSVDLYDEIEQIGSEKTQNVTVNPGPFAQTRYAPVNWGHGEINDSTTVEPILDGPYQPTTFTPPTDYWILINSNTNGVVYESTNNSDFWTAVIAVEPHVDPVDRQYNVFGENKQFNVRNDSDKWKFLEMFRGSSQSDFYNRRTLTSDTKLVGILKYGGRIWTFHGETPRATTDSSNTANLNGISITIHSEFYIIPRSQESKCNEYINNGLPPIQNTRNVVPLSLSSRSIQYTRAQVNEDITISKTSLWKEMQYNRDIIIRFKFGNSIIKLGGLGYKWSEISYKAANYQYNYLRDGEQVTAHTTCSVNGVNNFSYNGGSLPTDFSISRYEVIKENSYVYVDYWDDSKAFRNMVYVRSLAANLNSVKCTGGSYDFSIPVGAWPVMNGGAVSLHFAGVTLSTQFTDFVSLNSLRFRFSLTVDEPSFSILRTRTVNLYGLPAANPNNGNEYYEISGRFSLISLVPTNDDYQTPIMNSVTVRQDLERQLTDLREEFNSLSQEIAMSQLIDLALLPLDMFSMFSGIKSTIDLTKSMATSVMKKFRKSKLATSVSEMTNSLSDAASSASRSVSVRSNISAFSNWTNVSNDVSNVTNSVNDISTQTSTISKNLRLREMITQTEGMSFDDISAAVLKTKIDMSTQIGKNTLPDIVTEASEKFIPKRSYRILKDDEVMEINTEGKFFAYKIDTLNEVPFDVNKFAELVTNSPVISAIIDFKTLKNLNDNYGITRTEALNLIKSNPNVLRNFINQNNPIIRNRIEQLILQCKL

**VP6:**

>AIN41107.2 |inner capsid protein VP6 [Human rotavirus A]

MEVLYSLSKTLKDARDKIVEGTLYSNVSDLIQQFNQMIVTMNGNDFQTGGIGNLPIRNWTFDFGLLGTTLLNLDANYVETARTTIEYFIDFIDNVCMDEMARESQRNGVAPQSEALRKLAGIKFKRINFDNSSEYIENWNLQNRRQRTGFVFHKPNIFPYSASFTLNRSQPMHDNLMGTMWLNAGSEIQVAGFDYSCAINAPANIQQFEHIVQLRRALTTATITLLPDAERFSFPRVINSADGATTWFFNPVILRPNNVEVEFLLNGQIINTYQARFGTIIARNFDTIRLSFQLMRPPNMTPAVNALFPQAQPFQHHATVGLTLRIESAVCESVLADANETLLANVTAVRQEYAIPVGPVFPPGMNWTELITNYSPSREDNLQRVFTVASIRSMLIK

**VP7:**

>ADC32676.1 |VP7 protein [Human rotavirus G1P[8]]

MYGIEYTTILIFLISIILLNYILKSVTRIMDYIIYRFLLISVALFALTKAQNYGLNIPITGSMDTVYSNSTQEGIFLTSTLCLYYPTEASTQISDGEWKDSLSQMFLTKGWPTGSVYFKEYSNIVDFSVDPQLYCDYNLVLMKYDQNLELDMSELADLILNEWLCNPMDITLYYYQQSGESNKWISMGSSCTVKVCPLNTQTLGIGCQTTNVDSFETVAENEKLAIVDVVDGINHKINLTTTTCTIRNCKKLGPRENVAVIQVGGSNILDITADPTTNPQIERMMRVNWKRWWQVFYTIVDYINQIVQVMSKRSRSLNSAAFYYRV

>WDU50760.1 |capsid glycoprotein VP7 [Human rotavirus A G2]

MYGIEYTTILTILISIILLNYILKTITNTMDYIIFRFLLLIALMSPFVRTQNYGMYLPITGSLDAVYTNSTSGESFLTSTLCLYYPTEAKNEISDNEWENTLSQLFLTKGWPTGSVYFKDYNDITTFSMNPQLYCDYNVVLMRYDNTSELDASELADLILNEWLCNPMDISLYYYQQNSESNKWISMGTDCTVKVCPLNTQTLGIGCKTTDVDTFEIVASSEKLVITDVVNGVNHKINISINTCTIRNCNKLGPRENVAIIQVGGPNALDITADPTTVPQVQRIMRVNWKKWWQVFYTVVDYINQIIQVMSKRSRSLDTAAFYYRI

>YBQ72798.1 |VP7 [Rotavirus alphagastroenteritidis G3]

MYGIEYTTVLTFLISVILLNYVLKSLTRIMDFIIYRFLLIIVILSPLLNAQNYGINLPITGSMDTPYTNSTREEVFLTSTLCLYYPTEAATEINDNSWKDTLSQLFLIKGWPTGSIYFKDYTDIASFSVDPQLYCDYNLVLMKYDATLQLDMSELADLLLNEWLCNPMDITLYYYQQTDEANKWISMGSSCTIKVCPLNTQTLGIGCLTTDTNTFEEVATAEKLVITDVVDGVNHKLNVTTNTCTIRNCKKLGPRENVAVIQVGGPDVLDITADPTTMPQTERMMRVNWKKWWQVFYTIVDYVNQIVQAMSKRSRSLNSAAFYYRV

>ADT62510.1 |VP7 partial [Human rotavirus G9P[8]]

MYGIEYTTILTFLISIVLLNYILKSLTSAMDFIIYRFLLLIVIASPFVKTQNYGINLPITGSMDTAYANSSQQETFLTSTLCLYYPTEASTQIGDTEWKDTLSQLFLTKGWPTGSVYFKEYTDIASFSIDPQLYCDYNVVLMKYDSTLELDMSELADLILNEWLCNPMDITLYYYQQTDEANKWISMGQSCTIKVCPLNTQTLGIGCITTNTATFEEVATSEKLVITDVVDGVNHKLDVTTNTCTIRNCKKLGPRENVAIIQVGGSDVLDITADPTTAPQTERMMRVNWKKWWQVFYTVVDYINQIVQVMSKRSRSLNSAAFYYRV

>BAI49627.1 |outer capsid protein VP7 [Rotavirus G12]

MYGIEYTTILTFLISIILLNYILKSITNMMDFIIYRFLLIVVVILPFIKAQNYGINLPITGSMDTAYVNSTQQENFMTSTLCLYYPSSVTTEITDPDWTNTLSQLFLTKGWPTNSVYFKSYADISSFSVDPQLYCDYNIVLIQYQNSLALDVSELADLILNEWLCNPMDVTLYYYQQTDEANKWISMGESCTVKVCPLNTQTLGIGCTTTDVTTFEEVANAEKLVITDVVDGVNHKINITVNTCTIRNCKKLGPRENVAIIQVGSSDVIDITADPTTIPQTERMMRINWKKWWQVFYTVVDYINQIVQVMSKRSRSLNSAAFYYRI

**hexon:**

>WFP21729.1 |hexon [Human adenovirus 41]

MATPSMMPQWSYMHIAGQDASEYLSPGLVQFARATDTYFSLGNKFRNPTVAPTHDVTTDRSQRLTLRFVPVDREDTAYSYKVRFTLAVGDNRVLDMASTYFDIRGVLDRGPSFKPYSGTAYNSLAPKTAPNPCEWKDNNNKIKVRGQAPFIGTNIDKDKGIQIGTDTTNQPIYADKTYQPEPQVGQTQWNSEVGAAQKVAGRVLKDTTPMLPCYGSYAKPTNEKGGQASLITNGTDQTLTSDVNLQFFALPSTPNEPKAVLYAENVSIEAPDTHLVYKPDVAQGTISSADLLTQQAAPNRPNYIGFRDNFIGLMYYNSTGNMGVLAGQASQLNAVVDLQDRNTELSYQLMLDALGDRSRYFSMWNQAVDSYDPDVRIIENHGVEDELPNYCFPLGGSAATDTYSGIKANGQTWTADDNYADRGAEIESGNIFAMEINLAANLWRSFLYSNVALYLPDSYKITPDNITLPENKNTYAYMNGRVAVPSALDTYVNIGARWSPDPMDNVNPFNHHRNAGLRYRSMLLGNGRYVPFHIQVPQKFFAIKNLLLLPGSYTYEWNFRKDVNMILQSSLGNDLRVDGASVRFDSINLYANFFPMAHNTASTLEAMLRNDTNDQSFNDYLCAANMLYPIPSNATSVPISIPSRNWAAFRGWSFTRLKTKETPSLGSGFDPYFTYSGSVPYLDGTFYLNHTFKKVSIMFDSSVSWPGNDRLLTPNEFEIKRTVDGEGYNVAQCNMTKDWFLIQMLSHYNIGYQGFYVPESYKDRMYSFFRNFQPMSRQVVNTTTYKEYQNVTLPFQHNNSGFVGYMGPTMREGQAYPANYPYPLIGQTAVPSLTQKKFLCDRTMWRIPFSSNFMSMGALTDLGQNMLYANSAHALDMTFEVDPMDEPTLLYVLFEVFDVVRIHQPHRGVIEAVYLRTPFSAGNATT

**short fiber protein：**

>WOR09025.1 |short fiber protein [Human adenovirus 41]

MKRTRIEDDFNPVYPYDTFSTPSIPYVAPPFVSSDGLQEKPPGVLALKYTDPITTNAKHELTLKLGSNITLENGLLSATVPTVSPPLTNSNNSLGLATSAPIAVSANSLTLATAAPLTVSNNQLSINAGRGLVITNNAVAVNPTGALGFNNTGALQLNAAGGMRVDGANLILHVAYPFEAVNQLTLRLENGLEVTSGGKLNVKLGSGLQFDSNGRIAISNSNRTRSVPSLTTIWSISPTPNCSIYETQDANLFLCLTKNGAHVLGTITIKGLKGALREMHDNALSLKLPFDNQGNLLNCALESSTWRYQETDAVASNALTFMPNSTVYPRNKTADPGNMLIQISPNITFSVVYNEINSGYAFTFKWSAEPGKPFHPPTAVFCYITEQ

- 1. **Tetanus toxin immunodominant epitope P2 (GenBank: RXM90438.1)：**

QYIKANSKFIGITEL

- 1. **RS09**

APPHALS

**2.4 Vaccine sequence:**

MQYIKANSKFIGITELEAAAKAKLHRAGFMTVSSNTGGGGSIDFKTLKNLNDNYGIGGGGSWKEMQYNRDIIIRFKGGGGSEWNFRKDVNMILQSSGGGGSGSYTYEWNFRKDVNMAAAFVFVSWVSRAAAMSEVALVRYAAAKMRIVAMLYAAAIFAAVPPNFAAASQVTMFPHIAAALPILTLGELAAAIMNGGAVSLAAAYEIAGRFSLAAAILRTRTVNLAAAVMNGGAVSLAAARVFTVASIRAAANPMDITLYYAAATVVDYINQIAAASYKDRMYSFAAARSMLLGNGRAAATELSYQLMLKKHNSSQPQPTMRLVAMLKKGPELNPYLAHLARMYNKKGPDLNPYLSHLARMYNKKPGEMLLNLELGPELNPKKDITISKTSLWKEMQYNKKDFVSLNSLRFRFSLTVKKIDFKTLKNLNDNYGITKKNNVEVEFLLNGQIINTKKPMDITLYYYQQSGESNKKLMRYDNTSELDASELAKKEKLVITDVVNGVNHKIKKPTTIPQTERMMRINWKKKDRSQRLTLRFVPVDREKKPNYIGFRDNFIGLMYYKKNHHRNAGLRYRSMLLGKKTFMPNSTVYPRNKTADKKLIQISPNITFSVVYNEHHHHHH

- 1. **tPA sequence (UniProt ID: P00750):**

MDAMKRGLCCVLLLCGAVFVSPS

**2.6 MITD sequence (UniProt ID: Q8WV92):**

MAKSGLRQDPQSTAAATVLKRAVELDSESRYPQALVCYQEGIDLLLQVLKGTKDNTKRCNLREKISKYMDRAENIKKYLDQEKEDGKYHKQIKIEENATGFSYESLFREYLNETVTEVWIEDPYIRHTHQLYNFLRFCEMLIKRPCKVKTIHLLTSLDEGIEQVQQSRGLQEIEESLRSHGVLLEVQYSSSIHDREIRFNNGWMIKIGRGLDYFKKPQSRFSLGYCDFDLRPCHETTVDIFHKKHTKNI

**2.7 Kozak sequence:**

GCCATGATGG

**2.8 5’UTR:**

GGACAGATCGCCTGGAGACGCCATCCACGCTGTTTTGACCTCCATAGAAGACACCGGGACCGATCCAGCCTCCGCGGCCGGGAACGGTGCATTGGAACGCGGATTCCCCGTGCCAAGAGTGACTCACCGTCCTTGACACG

**2.9 3’UTR:**

TGACGGGTGGCATCCCTGTGACCCCTCCCCAGTGCCTCTCCTGGCCCTGGAAGTTGCCACTCCAGTGCCCACCAGCCTTGTCCTAATAAAATTAAGTTGCATCAAGCT

**2.10 The mRNA sequences of the designed vaccines:**

GGATCCGGACAGATCGCCTGGAGACGCCATCCACGCTGTTTTGACCTCCATAGAAGACACCGGGACCGATCCAGCCTCCGCGGCCGGGAACGGTGCATTGGAACGCGGATTCCCCGTGCCAAGAGTGACTCACCGTCCTTGACACGGCCATGATGGATGGACGCTATGAAACGTGGTCTGTGCTGCGTTCTGCTGCTGTGCGGTGCTGTTTTCGTTTCTCCGTCTGAAGCTGCTGCTAAAATGCAGTACATCAAAGCTAACTCTAAATTCATCGGTATCACCGAACTGGAAGCTGCTGCTAAAGCTAAACTCCACCGTGCTGGCTTCATGACCGTTTCGTCTAACACCGGTGGTGGTGGTTCTATCGACTTCAAAACCCTGAAAAACCTGAACGACAACTACGGTATCGGTGGTGGTGGTTCTTGGAAAGAAATGCAGTACAACCGTGACATCATCATCCGTTTCAAAGGTGGTGGTGGTTCTGAATGGAACTTCCGTAAAGACGTTAACATGATCCTGCAGTCTTCTGGTGGTGGTGGTTCTGGTTCTTACACCTACGAATGGAACTTCCGTAAAGACGTTAACATGGCTGCTGCTTTCGTTTTCGTTTCTTGGGTTTCTCGTGCTGCTGCTATGTCTGAAGTTGCTCTGGTTCGTTACGCTGCTGCTAAAATGCGTATCGTTGCTATGCTGTACGCTGCTGCTATCTTCGCTGCTGTTCCGCCGAACTTCGCTGCTGCTTCTCAGGTTACCATGTTCCCGCACATCGCTGCTGCTCTGCCGATCCTGACCCTGGGTGAACTGGCTGCTGCTATCATGAACGGTGGTGCTGTTTCTCTGGCTGCTGCTTACGAAATCGCTGGTCGTTTCTCTCTGGCTGCTGCTATCCTGCGTACCCGTACCGTTAACCTGGCTGCTGCTGTTATGAACGGTGGTGCTGTTTCTCTGGCTGCTGCTCGTGTTTTCACCGTTGCTTCTATCCGTGCTGCTGCTAACCCGATGGACATCACCCTGTACTACGCTGCTGCTACCGTTGTTGACTACATCAACCAGATCGCTGCTGCTTCTTACAAAGACCGTATGTACTCTTTCGCTGCTGCTCGTTCTATGCTGCTGGGTAACGGTCGTGCTGCTGCTACCGAACTGTCTTACCAGCTGATGCTGAAAAAACACAACTCTTCTCAGCCGCAGCCGACCATGCGTCTGGTTGCTATGCTGAAAAAAGGTCCGGAACTGAACCCGTACCTGGCTCACCTGGCTCGTATGTACAACAAAAAAGGTCCGGACCTGAACCCGTACCTGTCTCACCTGGCTCGTATGTACAACAAAAAACCGGGTGAAATGCTGCTGAACCTGGAACTGGGTCCGGAACTGAACCCGAAAAAAGACATCACCATCTCTAAAACCTCTCTGTGGAAAGAAATGCAGTACAACAAAAAAGACTTCGTTTCTCTGAACTCTCTGCGTTTCCGTTTCTCTCTGACCGTTAAAAAAATCGACTTCAAAACCCTGAAAAACCTGAACGACAACTACGGTATCACCAAAAAAAACAACGTTGAAGTTGAATTCCTGCTGAACGGTCAGATCATCAACACCAAAAAACCGATGGACATCACCCTGTACTACTACCAGCAGTCTGGTGAATCTAACAAAAAACTGATGCGTTACGACAACACCTCTGAACTGGACGCTTCTGAACTGGCTAAAAAAGAAAAACTGGTTATCACCGACGTTGTTAACGGTGTTAACCACAAAATCAAAAAACCGACCACCATCCCGCAGACCGAACGTATGATGCGTATCAACTGGAAAAAAAAAGACCGTTCTCAGAGGCTCACCCTGAGGTTCGTTCCGGTTGACCGTGAAAAAAAACCGAACTACATAGGCTTCCGTGACAACTTTATCGGTCTGATGTACTACAAAAAAAACCACCACCGTAACGCTGGTCTGCGTTACCGTTCTATGCTGCTGGGTAAAAAAACCTTCATGCCGAACTCTACCGTTTACCCGCGTAACAAAACCGCTGACAAAAAACTGATCCAGATCTCTCCGAACATCACCTTCTCTGTTGTTTACAACGAACACCACCACCACCACCACGAAGCTGCTGCTAAAATGGCTAAATCTGGTCTGCGTCAGGACCCGCAGTCTACCGCTGCTGCTACCGTTCTGAAACGTGCTGTTGAACTGGACTCTGAATCTCGTTACCCGCAGGCTCTGGTTTGCTACCAGGAAGGTATCGACCTGCTGCTGCAGGTTCTGAAAGGTACCAAAGACAACACCAAACGTTGCAACCTGCGTGAAAAAATCTCTAAATACATGGACCGTGCTGAAAACATCAAAAAATACCTGGACCAGGAAAAAGAAGACGGTAAATACCACAAACAGATCAAAATCGAAGAAAACGCTACCGGTTTCTCTTACGAATCTCTGTTCCGTGAATACCTGAACGAAACCGTTACCGAAGTTTGGATCGAAGACCCGTACATCCGTCACACCCACCAGCTGTACAACTTCCTGCGTTTCTGCGAAATGCTGATCAAACGTCCGTGCAAAGTTAAAACCATCCACCTGCTGACCTCTCTGGACGAAGGTATCGAACAGGTTCAGCAGTCTCGTGGTCTGCAGGAAATCGAAGAATCTCTGCGTTCTCACGGTGTTCTGCTGGAAGTTCAGTACTCTTCTTCTATCCACGACCGTGAAATCCGTTTCAACAACGGTTGGATGATCAAAATCGGTCGTGGTCTGGACTACTTCAAAAAACCGCAGTCTCGTTTCTCTCTGGGTTACTGCGACTTCGACCTGCGTCCGTGCCACGAAACCACCGTTGACATCTTCCACAAAAAACACACCAAAAACATCTAATGACGGGTGGCATCCCTGTGACCCCTCCCCAGTGCCTCTCCTGGCCCTGGAAGTTGCCACTCCAGTGCCCACCAGCCTTGTCCTAATAAAATTAAGTTGCATCAAGCTCTCGAG

1. **Supplementary Table**

**3.1 Supplementary Table 1** Comprehensive bioinformatics parameters for the whole process of trivalent multi-epitope mRNA vaccine development.

| **Primary Theme** | | **Secondary Theme** | **Server/Software** | **Specific Parameters** |
| --- | --- | --- | --- | --- |
| 1. Target Protein Screening | | Ref Seq Retrieval | NCBI Virus Database | Initial lib: 3 norovirus + 11 rotavirus + 25 adenovirus 40/41 proteins |
| 2. Target Protein Screening | | Antigenicity | VaxiJen v2.0 | Score > 0.4 (retained) |
|  | | Safety (Toxic/Allergenic) | ToxinPred v2.0 & AllerTOP v2.0 | ToxinPred v2.0 >0.6 OR AllerTOP >0.5 (excluded) |
|  | | Expression Difficulty | DeepTMHMM v1.0 & SignalP-6.0 | TMHMM: >1 transmembrane helix (excluded) / SignalP: signal peptide (excluded) |
|  | | Subcellular Localization | Virus-mPLoc | Viral capsid surface/ Viral membrane surface / Extracellular region |
|  | | Phylogenetic tree | CD-HIT; MAFFT v7.407; IQ-TREE v2; iTOL | Dedup; length ≥95% ref; human only; remove “X”; CD-HIT: 100% identity (de-redundancy)  MAFFT: L-INS-i; maxiterate=1000; IQ-TREE: maximum-likelihood; ModelFinder (auto model selection); UFBoot=1000; Visualization: iTOL |
| 3. Epitope Prediction | | CTL HLA Binding | IEDB (NetMHCpan 4.1) | Peptide: 9 aa; 27 HLA alleles; BA affinity <50 nM (retained); EL percentile <0.5% |
|  | | CTL Processing | IEDB (MHC-I Processing Tool) | Comprehensive score >0 (retained) |
|  | | CTL Safety | VaxiJen v2.0, Algpred v2.0, ToxinPred v3.0 | VaxiJen >0.4; Algpred <0.3; ToxinPred <0.38 |
|  | | HTL HLA Binding | IEDB (MHC II Tool) | Peptide: 15 aa; 27 HLA alleles; BA affinity <50 nM; EL percentile <2% |
|  | | HTL Cytokine Induction | IFNepitope, IL4pred | IFNepitope >0.3; IL4pred >0.2 |
|  | | Linear B-Cell Epitope | ABCpred server | 16 aa windows; threshold 0.51; same as T-cell (safety) |
|  | | Secondary validation | MixMHCpred v3.0;  MixMHC2pred v2.0.2 | %Rank_bestAllele <0.5% + positive Score_bestAllele  %Rank_best <1% + SubSpec_best=1 |
|  | | Epitope Conservancy | IEDB (Conservancy Tool) | Identity >90% |
| 4. Vaccine Design | | Sequence Construction | NA | Linkers: CTL-CTL=AAA; HTL-HTL =GGGGS; LBL-LBL=KK; P2 adjuvant linked via EAAAK; 6×His tag (C-term) |
|  | | Physicochemical Property | ProtParam, Protein-Sol | ProtParam: Instability index <40; Protein-Sol: Solubility evaluation |
|  | Structure Prediction | | PSIPRED4.0, Protenix, AlphaFold3/2, Chai1, GalaxyWEB | PSIPRED: secondary; Protenix, AlphaFold3/2, Chai1: Tertiary structure; GalaxyWEB: Tertiary structure optimization. |
|  | Structure Quality | | SAVES v6.1, PROCHECK, ProSA-web | SAVES: ERRAT >85; PROCHECK: ≥90% in favored regions |
|  | Epitope-HLA Docking | | RCSB PDB, SWISS-MODEL, HPEPDOCK2.0, PyMOL v3.1, LigPlot+ | SWISS-MODEL: >80% coverage, GMQE >0.7; LigPlot+: H-bond <3.5 Å |
| 5. Molecular Docking | Vaccine-TLR2/TLR4/HLA Docking | | RCSB PDB, ClusPro 2.0, HADDOCK 2.4, PDBsum | TLR2 (PDB 2Z7X); TLR4 (PDB 8WO1); ClusPro: top 3 conformations; HADDOCK: default opt |
|  | Simulation Setup | | Gromacs 2023.3 | Force field: Amber99SB-ILDN; Water: TIP3P; 0.15 M NaCl; 100 ns (2 fs step) |
| 6. Molecular Dynamics | Result Analysis | | Gromacs 2023.3, GMX_MMPBSA v1.62 | RMSD <2 Å; RMSF <2 Å; H-bond ≥3/frame; MM-PBSA (last 10 ns) |
| 7. Immune & Coverage | Immune Simulation | | C-ImmSim server | Three injections at days 1/28/56 (default simulator units) |
|  | Population Coverage | | IEDB (Coverage Tool) | Input CTL/HTL + HLA; coverage: Europe/Asia/Africa/Americas/Oceania |
| 8. mRNA Vaccine Assembly | Sequence Assembly | | NA | 5': Kozak (GCCACC) + tPA + EAAAK; 3': EAAAK + MITD + TAA + hGH 3'UTR (100 bp); sites: BamHI (GGATCC) & XhoI (CTCGAG) |
|  | Secondary Structure | | RNAfold server | ΔG > -30 kcal/mol (low interference) |
|  | Codon Optimization | | Jcat server | Host: E. coli K12; GC 40-60%; CAI >0.9; rare codons excluded |
|  | Vector Cloning | | GenSmart™ Design server | Vector: pET-28a (+); insertion: N-BamHI, C-XhoI |

| **3.2 Supplementary Table 2** BLASTp Results of CTL Epitopes. | | | | | | | |
| --- | --- | --- | --- | --- | --- | --- | --- |
| **protein** | **subtype** | | **Sequence** | **E value** | **Query Coverage** | **Identity** | **E value of Human homology** |
| **VP1** | GI.6 | | FVFVSWVSR | 3.00E-07 | 100% | 100.00% | 34 |
|  | GII.2 | | MSEVALVRY | 8.00E-06 | 100% | 100.00% | 69 |
|  |  | | KMRIVAMLY | 1.00E-06 | 100% | 100.00% | 39 |
|  | GII.4 | | IFAAVPPNF | 9.00E-05 | 100% | 100.00% | 27 |
|  |  | | SQVTMFPHI | 2.00E-05 | 100% | 100.00% | 39 |
|  | GII.6 | | LPILTLGEL | 2.00E-05 | 100% | 100.00% | 55 |
| **VP4** | P4 | | IMNGGAVSL | 2.00E-04 | 100% | 100.00% | 27 |
|  | P6 | | YEIAGRFSL | 9.00E-05 | 100% | 100.00% | 69 |
|  | P8 | | ILRTRTVNL | 1.00E-04 | 100% | 100.00% | 48 |
|  |  | | VMNGGAVSL | 4.00E-04 | 100% | 100.00% | 12 |
| **VP6** | A | | RVFTVASIR | 2.00E-04 | 100% | 100.00% | 12 |
| **VP7** | G1\G3\G9 | | NPMDITLYY | 6.00E-07 | 100% | 100.00% | 34 |
|  | G2 | | TVVDYINQI | 5.00E-06 | 100% | 100.00% | 24 |
| **hexon** | 41\41 | | SYKDRMYSF | 2.00E-06 | 100% | 100.00% | 24 |
|  |  | | RSMLLGNGR | 3.00E-05 | 100% | 100.00% | 139 |
|  | |  | TELSYQLML | 8.00E-06 | 100% | 100.00% | 34 |

| **3.3 Supplementary Table 3** BLASTp Results of HTL Epitopes. | | | | | | |
| --- | --- | --- | --- | --- | --- | --- |
| **protein** | **subtype** | **Sequence** | **E value** | **Query Coverage** | **Identity** | **E value of Human homology** |
| **VP1** | GII.2 | AKLHRAGFMTVSSNT | 2.00E-12 | 100% | 100% | 37 |
| **VP4** | P4/P6/P8 | IDFKTLKNLNDNYGI | 2.00E-11 | 100% | 100% | 26 |
|  | P4/P8 | WKEMQYNRDIIIRFK | 2.00E-13 | 100% | 100% | 26 |
| **hexon** | 40/41 | EWNFRKDVNMILQSS | 6.00E-14 | 100% | 100% | 18 |
|  |  | GSYTYEWNFRKDVNM | 1.00E-14 | 100% | 100% | 37 |

| **3.4 Supplementary Table 4** BLASTp Results of LBL Epitopes. | | | | | | | |  |
| --- | --- | --- | --- | --- | --- | --- | --- | --- |
| **protein** | **subtype** | | **Sequence** | **E value** | **Query Coverage** | **Identity** | **E value of Human homology** | |
| **VP1** | GI.6 | | HNSSQPQPTMRLVAML | 4.00E-15 | 100% | 100% | 22 | |
|  | GII.2 | | GPELNPYLAHLARMYN | 5.00E-15 | 100% | 100% | 15 | |
|  | GII.4 | | GPDLNPYLSHLARMYN | 2.00E-14 | 100% | 100% | 43 | |
|  | GII.6 | | PGEMLLNLELGPELNP | 2.00E-14 | 100% | 100% | 22 | |
| **VP4** | P4/P8 | | DITISKTSLWKEMQYN | 2.00E-13 | 100% | 100% | 11 | |
|  | P4/P6/P8 | | DFVSLNSLRFRFSLTV | 7.00E-12 | 100% | 100% | 31 | |
|  |  | | IDFKTLKNLNDNYGIT | 2.00E-12 | 100% | 100% | 30 | |
| **VP6** | A | | NNVEVEFLLNGQIINT | 2.00E-12 | 100% | 100% | 11 | |
| **VP7** | G1 | | PMDITLYYYQQSGESN | 5.00E-15 | 100% | 100% | 11 | |
|  | G2 | | LMRYDNTSELDASELA | 5.00E-14 | 100% | 100% | 22 | |
|  |  | | EKLVITDVVNGVNHKI | 9.00E-14 | 100% | 100% | 124 | |
|  | G12 | | PTTIPQTERMMRINWK | 3.00E-13 | 100% | 100% | 61 | |
| **hexon** | 40/41 | | DRSQRLTLRFVPVDRE | 2.00E-14 | 100% | 100% | 61 | |
|  |  | | PNYIGFRDNFIGLMYY | 1.00E-15 | 100% | 100% | 30 | |
|  |  | | NHHRNAGLRYRSMLLG | 3.00E-14 | 100% | 100% | 43 | |
| **short fiber protein** | 40/41 | | TFMPNSTVYPRNKTAD | 3.00E-14 | 100% | 100% | 15 | |
|  | |  | LIQISPNITFSVVYNE | 3.00E-14 | 100% | 100% | 11 | |

| **3.5 Supplementary Table 5** National-Level Population Coverage. | | | |
| --- | --- | --- | --- |
| population/area | Class combined | | |
|  | coverage | average_hit | pc90 |
| Sweden | 100.00% | 4.71 | 3.31 |
| United States | 99.99% | 3.67 | 2.37 |
| Peru | 99.98% | 2.9 | 1.53 |
| Mexico | 99.93% | 3.82 | 2.45 |
| France | 99.92% | 3.56 | 2.38 |
| Finland | 99.83% | 3.57 | 2.25 |
| Russia | 99.77% | 3.76 | 2.33 |
| Brazil | 99.40% | 3.26 | 2.03 |
| Austria | 99.17% | 2.81 | 1.78 |
| Germany | 99.10% | 3.01 | 1.85 |
| England | 99.00% | 2.92 | 1.77 |
| Ireland Northern | 98.86% | 2.86 | 1.73 |
| Papua New Guinea | 98.84% | 2.81 | 1.44 |
| Ireland South | 98.82% | 2.84 | 1.73 |
| Spain | 98.81% | 2.35 | 1.37 |
| Uganda | 98.15% | 2.94 | 1.68 |
| Belgium | 97.92% | 2.56 | 1.37 |
| Cameroon | 97.86% | 2.8 | 1.54 |
| Poland | 97.51% | 2.5 | 1.38 |
| Slovenia | 97.45% | 1.12 | 1.09 |
| Kenya | 97.37% | 2.62 | 1.47 |
| Zambia | 97.25% | 2.3 | 1.31 |
| Japan | 97.08% | 3.43 | 1.89 |
| Argentina | 97.04% | 2.98 | 1.45 |
| Croatia | 96.80% | 2.47 | 1.33 |
| Italy | 96.62% | 2.48 | 1.37 |
| Czech Republic | 96.46% | 2.27 | 1.27 |
| Cape Verde | 96.38% | 2.54 | 1.37 |
| India | 96.18% | 2.58 | 1.27 |
| Romania | 95.81% | 2.32 | 1.25 |
| Saudi Arabia | 95.52% | 2.59 | 1.28 |
| West Indies | 95.20% | 2.44 | 1.26 |
| Cuba | 94.99% | 2.4 | 1.24 |
| Mali | 94.93% | 2.35 | 1.26 |
| Sao Tome and Principe | 94.56% | 2.31 | 1.21 |
| Tunisia | 94.49% | 2.22 | 1.19 |
| Morocco | 94.18% | 2.09 | 1.15 |
| China | 93.21% | 2.53 | 1.15 |
| American Samoa | 92.92% | 2.94 | 1.44 |
| New Caledonia | 92.92% | 2.49 | 1.97 |
| Tokelau | 92.86% | 0.95 | 1.03 |
| Portugal | 92.82% | 2.13 | 1.1 |
| Georgia | 92.51% | 2.21 | 1.1 |
| Guinea-Bissau | 92.34% | 2.18 | 1.1 |
| Senegal | 92.21% | 2.2 | 1.1 |
| Philippines | 92.04% | 2.36 | 1.1 |
| Mongolia | 91.67% | 2.2 | 1.07 |
| Zimbabwe | 91.37% | 1.92 | 1.05 |
| Venezuela | 90.55% | 1.89 | 1.02 |
| Bulgaria | 90.54% | 1.88 | 1.02 |
| Ecuador | 90.20% | 2.26 | 1.01 |

|  | **3.6 Supplementary Table 6** List of Bioinformatics Software/Servers Used, Their Versions, Affiliated Institutions and Countries. | | | |
| --- | --- | --- | --- | --- |
|  | Software or server | Version | Main Affiliated Institutions | Country |
|  | The NCBI Virus Database | continuously updated | National Center for Biotechnology Information (NCBI) | United States of America |
|  | The VaxiJen v2.0 server | V2.0 | Developed by Doytchinova & Flower; originally hosted at Jenner Institute (UK); currently available at ddg-pharmfac.net (Drug Design Group) | United Kingdom (original) / Bulgaria (current hosting) |
|  | The ToxinPred v2.0 server | V2.0 | IIIT Delhi/Indraprastha Institute of Information Technology, Delhi | India |
|  | The AllerTOP v2.0 server | V2.0 | Drug Design and Bioinformatics Lab, Faculty of Pharmacy, Medical University of Sofia | Bulgaria |
|  | The DeepTMHMM server | V1.0 | Technical University of Denmark | Denmark |
|  | The Virus-mPLoc server | V1.0 | Shanghai Jiaotong University | China |
|  | MAFFT v7.407 | V7.407 | The National Institute of Advanced Industrial Science and Technology ;Osaka University | Japan |
|  | the iTOL server | V7.4 | Biobyte solutions GmbH；European Molecular Biology Laboratory | Germany |
|  | the MHC Class I Tools Suite | Continuously updated | La Jolla Institute for Allergy and Immunology | United States of America |
|  | the MHC Class II Tools Suite | Continuously updated | La Jolla Institute for Allergy and Immunology | United States of America |
|  | the Algpred v2.0 | V2.0 | Department of Computational Biology, Indraprastha Institute of Information Technology (IIIT-Delhi) | India |
|  | the ToxinPred v3.0 | V3.0 | Department of Computational Biology, Indraprastha Institute of Information Technology (IIIT-Delhi) | India |
|  | the IFNepitope server | V1.0 | Department of Computational Biology Indraprastha Institute of Information Technology Okhla Phase 3, New Delhi, INDIA | India |
|  | the IL4pred server | V1.0 | Department of Computational Biology Indraprastha Institute of Information Technology New Delhi | India |
|  | the ABCpred server | V1.0 | Department of Computational Biology, Indraprastha Institute of Information Technology (IIIT-Delhi) | India |
|  | the MixMHCpred v3.0 server | V3.0 | Department of Oncology, Ludwig Institute for Cancer Research Lausanne, University of Lausanne, Switzerland | Switzerland |
|  | the MixMHC2pred v2.0.2 server | V2.0.2 | Department of Oncology, Ludwig Institute for Cancer Research Lausanne, University of Lausanne, Switzerland | Switzerland |
|  | the IEDB Epitope Conservancy Analysis Tool | Continuously updated | IEDB Analysis Resource / IEDB | United States of America |
|  | the NCBI BLASTp server | V2.17.0 | National Center for Biotechnology Information | United States of America |
|  | the RCSB Protein Data Bank | continuously updated | RCSB PDB is managed by RCSB PDB consortium (Rutgers + UCSD/SDSC + UCSF) | United States of America |
|  | the SWISS-MODEL server | continuously updated | The Computational Structural Biology Group at the SIB Swiss Institute of Bioinformatics and the Biozentrum of the University of Basel | Switzerland |
|  | the HPEPDOCK2.0 server | V2.0 | the Huang Lab (Sheng-You Huang's research group) at the Institute of Biophysics, School of Physics, Huazhong University of Science and Technology | China |
|  | PyMOL v3.1 | V3.1 | Schrödinger, Inc. | United States of America |
|  | LigPlot+ tool | V2.2.9 | the Thornton Group at the EMBL-European Bioinformatics Institute (EMBL-EBI) | United Kingdom |
|  | the ProtParam server | Continuously updated | the SIB Swiss Institute of Bioinformatics | Switzerland |
|  | the SignalP-6.0 server | V6.0 | the Department of Health Technology, Technical University of Denmark | Denmark |
|  | the Protein-Sol server | Continuously updated | the Warwicker group at the University of Manchester | United Kingdom |
|  | the PSIPRED4.0 server | PSIPRED4.0 | the UCL Bioinformatics Group | United Kingdom |
|  | the Protenix server | Continuously updated | ByteDance AML AI4Science Team / ByteDance Seed | China |
|  | the AlphaFold3 server | V3.0 | Google DeepMind and Isomorphic Labs | United Kingdom |
|  | the AlphaFold2 server | V2.0 | Google DeepMind (a subsidiary of Alphabet Inc.), UK. | United Kingdom |
|  | the Chai1 server | V1.0 | Chai Discovery | United States of America |
|  | the GalaxyWEB server | V1.0 | Computational Biology Lab, Department of Chemistry, Seoul National University | Republic of Korea |
|  | the SAVES v6.1 server | V6.1 | UCLA-DOE LAB (Department of Energy Laboratory at University of California, Los Angeles) | United States of America |
|  | the ProSA-web server | continuously updated | CAME (Computational Applied Molecular Evolution) Group, University of Salzburg | Austria |
|  | the ClusPro 2.0 server | V2.0 | Boston University (BU) | United States of America |
|  | the HADDOCK 2.4 server | V2.4 | Utrecht University | Kingdom of the Netherlands |
|  | the PDBsum server | V1.0 | European Molecular Biology Laboratory, European Bioinformatics Institute (EMBL-EBI), Wellcome Trust Genome Campus, Hinxton | United Kingdom |
|  | the Gromacs 2023.3 software | V2023.3 | GROMACS development team (international) | International collaboration (primarily Netherlands, Sweden) |
|  | the GMX_MMPBSA v1.62 | V1.62 | Faculty of Basic Sciences, University of Medellin (Colombia) | Colombia |
|  | the C-ImmSim server | Continuously updated | Istituto per le Applicazioni del Calcolo "Mauro Picone" (IAC), National Research Council (CNR) of Italy, Rome | Italy |
|  | the Population Coverage analysis tool | Continuously updated | La Jolla Institute for Allergy and Immunology | United States of America |
|  | the Jcat server | Continuously updated | Institute of Microbiology, Technische Universität Braunschweig (Braunschweig University of Technology) | Germany |
|  | the RNAfold server | V2.6.4 | Institute for Theoretical Chemistry, University of Vienna | Austria |
|  | the GenSmart platform | Continuously updated | GenScript Biotech Corporation | United States (headquarters) and China (major R&D and operational base) |
